# Milestones in the development of *Myxococcus xanthus* as a model multicellular bacterium

**DOI:** 10.1128/jb.00071-25

**Published:** 2025-06-17

**Authors:** Lee Kroos, Daniel Wall, Salim T. Islam, David E. Whitworth, José Muñoz-Dorado, Penelope I. Higgs, Mitchell Singer, Emilia M.F. Mauriello, Anke Treuner-Lange, Lotte Søgaard-Andersen, Christine Kaimer, Montserrat Elías-Arnanz, Emina A. Stojković, Rolf Müller, Carsten Volz, Gregory J. Velicer, Beiyan Nan

**Affiliations:** 1 Department of Biochemistry and Molecular Biology, Michigan State University 3078, East Lansing, Michigan, USA; 2 Department of Molecular Biology, University of Wyoming 173150, Laramie, Wyoming, USA; 3 Institut National de la Recherche Scientifique (INRS), Centre Armand-Frappier Santé Biotechnologie, Institut Pasteur International Network 14851, Laval, Québec, Canada; 4 PROTEO, The Quebec Network for Research on Protein Function, Engineering, and Applications, Université de Montréal 5622, Montreal, Québec, Canada; 5 Department of Life Sciences, Aberystwyth University 1026, Aberystwyth, Wales, United Kingdom; 6 Departamento de Microbiología, Facultad de Ciencias, Universidad de Granada 117396 , Granada, Andalusia, Spain; 7 Department of Biological Sciences, Wayne State University 462768, Detroit, Michigan, USA; 8 Department of Microbiology and Molecular Genetics, University of California Davis 200415, Davis, California, USA; 9 Laboratoire de Chimie Bactérienne, CNRS 129862, Marseille, Provence-Alpes-Côte d'Azur, France; 10 Department of Ecophysiology, Max Planck Institute for Terrestrial Microbiology 28310, Marburg, Hesse, Germany; 11 Department of Biology and Biotechnology, Microbiology, Ruhr University Bochum 9142, Bochum, North Rhine-Westphalia, Germany; 12 Departamento de Genética y Microbiología, Área de Genética (Unidad Asociada al IQF CSIC), Facultad de Biologia, Universidad de Murcia 98701, Murcia, Region of Murcia, Spain; 13 Department of Biology, Northeastern Illinois University 466222, Chicago, Illinois, USA; 14 Department of Microbial Natural Products (MINS), Helmholtz Institute for Pharmaceutical Research Saarland (HIPS), Helmholtz Centre for Infection Research (HZI) and Department of Pharmacy at Saarland University 443745, Saarbrücken, Saarland, Germany; 15 German Center for Infection Research (DZIF), Braunschweig, Germany; 16 Institute for Integrative Biology, ETH Zurich 27219, Zürich, Zurich, Switzerland,; 17 Department of Biology, Texas A&M University 14736, College Station, Texas, USA; Geisel School of Medicine at Dartmouth, Hanover, New Hampshire, USA

**Keywords:** myxobacteria, evolution, multicellularity, sporulation, type 4 pilus, gliding motility, signal transduction, predation, secondary metabolites, microbial ecology, peptidoglycan, kin recognition, cell polarity, polysaccharides, cell division, photoreception

## Abstract

From the humblest of beginnings (i.e. a pile of dry cow dung) over 80 years ago, the Gram-negative bacterium *Myxococcus xanthus* has emerged as a premier model system for studying diverse fields of bacteriology, including multicellular development, sporulation, motility, cell-envelope biogenesis, spatiotemporal regulation, signaling, photoreception, kin recognition, social evolution, and predation. As the flagship representative of myxobacteria found in varied terrestrial and aquatic environments, *M. xanthus* research has evolved into a collaborative global effort, as reflected by the contributions to this article. In celebration of the upcoming 50th anniversary of the International Conference on the Biology of Myxobacteria, this review highlights the historical and ongoing contributions of *M. xanthus* as a multifaceted model bacterium.

## INTRODUCTION

## MYXOBACTERIAL RESEARCH: EARLY DAYS AND PIONEERING WAYS

Myxobacteria are diverse, rod-shaped Gram-negative bacteria found in terrestrial and aquatic environments worldwide (1). A major claim to fame of these microorganisms is that the life cycle of myxobacteria comprises two stages that depend on nutrient availability. When nutrients are abundant, cells follow a vegetative cycle, producing dynamic swarms; however, upon starvation, cells initiate a developmental cycle, culminating in myxospore-filled fruiting bodies.

**Fig 1:**
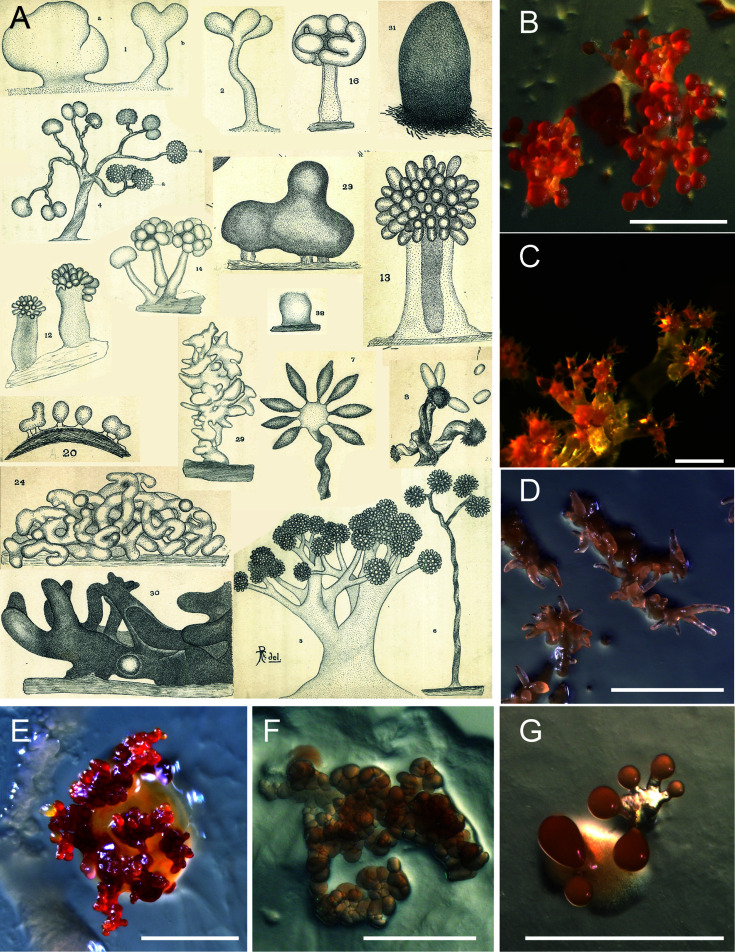
Myxobacteria in various fruiting-body complexities. (**A**) Fruiting bodies of diverse myxobacterial species, as sketched by Roland Thaxter, 1892 (3). Used with permission from the Archives of the Farlow Herbarium of Cryptogamic Botany, Harvard University, Cambridge, MA, USA. (**B**) *Stigmatella aurantiaca*, (**C**) *Chondromyces lanuginosus*, (**D**) *Corallococcus coralloides*, (**E**) *Cystobacter velatus*, (**F**) *Polyangium spomosum*, and (**G**) *Myxococcus stipitatus*. Photo credit of B–G: Ronald Garcia/HIPS. Scale bars, 500 µm**.**

Long assigned to the phylum δ-proteobacteria, myxobacteria have recently been reclassified into the newly established phylum Myxococcota (2). With a name derived from the Greek word “*myxa*” meaning “slime” or “mucus,” myxobacteria were originally thought to be related to slime molds and were mistakenly first classified as part of the deuteromycetes (i.e., *fungi imperfecti*). However, seminal work from the American mycologist, botanist, and entomologist Roland Thaxter in 1892 correctly identified these microorganisms as bacteria, describing the formation of pigmented, macroscopic, multicellular spore-filled fruiting bodies embedded in a slime matrix (Fig. 1). From this pioneering work, multiple groups of myxobacteria were identified, which included rod-shaped cells that transformed into circular spores and consequently were given the name “*Myxococcus*” to reflect this morphological transition (3).

Interest in morphological and cytological studies of myxobacteria then began to blossom toward the first half of the 20th century. During this period, a previously undescribed species of motile *Myxococcus* was isolated by J. M. Beebe, growing on dry cow dung in a pasture near Ames, Iowa (USA). Due to the yellow pigmentation of its fruiting bodies and vegetative swarms, the species was given the name “*xanthus*” (derived from the Greek word “*xanthós*” meaning “yellow” or “golden”), thus yielding *Myxococcus xanthus* (4).

In the 1960s and 1970s, Hans Reichenbach’s photos and time-lapse videos of myxobacteria revealed their beauty, diversity, and complexity, attracting investigators (5). Martin Dworkin’s fascination with the *M. xanthus* developmental cycle led to seminal publications on the nutritional regulation of multicellular fruiting-body morphogenesis (6) and the discovery of chemically induced unicellular sporulation (7). He passionately championed *M. xanthus* as a model organism and was joined by Dale Kaiser, whose sabbatical with Dworkin culminated in a method to transfer genes from *Escherichia coli* to *M. xanthus* using phage P1 (8). Eugene Rosenberg and David Zusman were also early leaders of the *M. xanthus* field, publishing a series of papers on DNA, RNA, and protein synthesis during vegetative growth (9), and in parallel with the Dworkin group, on DNA synthesis during chemically induced sporulation (10, 11) and germination (12). The Zusman group at the University of California, Berkeley, and the Kaiser group at Stanford University isolated generalized transducing phages, allowing transfer of genetic markers between *M. xanthus* strains (13, 14). Most researchers across the globe now studying *M. xanthus* (and most of the authors of this review) were either trained directly in the Kaiser or Zusman labs, or by their scientific progeny.

Key insights and new methods began to rapidly shape the *M. xanthus* field. The first book on myxobacteria (myxo-book) in 1984 (15) reflected the heavy emphasis on *M. xanthus* development, motility, and genetics at the time. It is here that this model system first received some of its most enduring praise when Rosenberg described the bacterium as having “become the *Escherichia coli* of Developmental Biology.” Upon completion of the second myxo-book in 1993 (16), a vibrant research community had emerged, and *M. xanthus* had become an invaluable model for studying additional fundamental cellular processes shared across the bacterial kingdom, including metabolism, gene regulation, signal transduction, and cell–cell communication. Since then, two more myxo-books have been published (17, 18), and many new research topics have emerged, such as cell-polarity regulation, cell-wall dynamics, polysaccharide secretion, chromosome segregation and cell division, photoreception, predation, cooperation and cheating, kin discrimination, and evolution.

Research on *M. xanthus* as a model organism continues to yield surprises and exciting discoveries of broad relevance to the bacterial kingdom. In this review, we discuss the history of major research topics using *M. xanthus* and related myxobacteria as model organisms.

## FROM LEA TO LAB: ORIGINS OF *M. XANTHUS* LABORATORY STRAINS

The founding *M. xanthus* isolate from Beebe was transferred to the strain collection of Roger Stanier at the University of California, Berkeley, which housed other *M. xanthus* strains such as the now widely used wild-type laboratory strain DZ2 (capable of single-cell and swarm motility). The “Beebe” strain was also deposited with the American Type Culture Collection (strain 19368); however, in the 1960s, this strain could no longer be revived by staff at the ATCC, leading to a request to Martin Dworkin to deposit his strain of *M. xanthus* FB (ATCC 25232) as it was believed to be the same as ATCC 19368. Incidentally, “FB” turned out to be a mixture of related variants that diverged from a common ancestor through extended propagation in the laboratory. Single-colony isolates from “FB” yielded strains DK101 and YS (both deficient in swarm spreading but not single-cell motility). DK101 was then subjected to UV mutagenesis, yielding the non-motile strain DK320. Subsequent rounds of transduction using the Mx8 phage and strain YS donor DNA resulted in strain DK1622, akin to a restored wild-type strain once again capable of single-cell and swarm-level motility. As DK1622 was the first myxobacterial strain to have its chromosome sequenced (19), this wealth of unlocked genetic information led to the widespread adoption of this strain for *M. xanthus* research. The genome of strain DZ2 was later found to be identical to that of DK1622, except for 196 kb of prophage-like DNA present in the former but not the latter (20). Together, strains DK1622 and DZ2 have become the workhorses of genetically tractable *M. xanthus* research in laboratories across the globe, with early work using those strains focusing on deciphering the molecular genetics of fruiting-body formation.

## FROM CODE TO COMPLEXITY: MYXOBACTERIAL GENOMICS

In general, myxobacteria have extremely large genomes for bacteria, containing an unusually high proportion (~70%) of GC base pairs. Prior to the current-day genomics revolution, it became apparent in the late 1990s and 2000s that whole-genome sequencing for bacteria was possible, and the myxobacterial research community was galvanized to unite and conduct large-scale projects. The size and high GC content of the *M. xanthus* genome made it an ambitious target for sequencing at the time, resulting in collaboration between the research community, the Monsanto company, and The Institute for Genomic Research (TIGR).

The genome sequence of *M. xanthus* DK1622 (9.14 Mbp) was first published in 2006 (19) and is typical of myxobacterial genomes. They comprise a single large circular chromosome, and while plasmids have been occasionally found in certain strains, they are lacking in most. Long-range synteny (conservation of gene order) is observed, even when comparing sister genera, and they are notable for their large numbers of biosynthetic gene clusters for producing secondary metabolites (representing at least 8.6% of the DK1622 genome). The large size of myxobacterial genomes was initially thought to be due to lineage-specific gene duplications, especially for genes encoding signaling proteins and transcriptional regulators (19). However, it now seems more likely that many of the “extra” genes were instead acquired by horizontal gene transfer (HGT) (21).

Another landmark myxobacterial genome sequence was reported soon thereafter in 2007, resulting from a community collaboration. At 13.03 Mbp, the *Sorangium cellulosum* So ce56 genome is unusually large, even for a myxobacterium, and it remains one of the largest bacterial genomes to have been completely sequenced (22). In contrast*, Anaeromyxobacter dehalogenans* 2CP-C belongs to an unusual myxobacterial genus with atypically small genomes of ~5 Mbp; incidentally, while not the first published, the *A. dehalogenans* 2CP-C genome was the first myxobacterial genome made publicly available via GenBank in early 2006 (23). The availability of genome sequences from multiple myxobacteria was transformative, enabling diverse downstream investigations utilizing reverse genetics, comparative genomics, functional genomics, transcriptomics, and proteomics, as well as community-wide microarray projects for *M. xanthus* DK1622 (24).

Nowadays, the accessibility of genome sequencing and post-genomic approaches has been particularly exploited by research groups studying ecological, environmental, secondary-metabolite, and/or taxonomic aspects of myxobacterial biology. For instance, genome sequencing has shown how predators and prey coevolve (25), allowed the construction of metagenome-assembled genomes of environmental myxobacteria (26), and provided useful information for defining taxa and assigning strains therein (21).

Comparing the genomes of strains within taxa has recently allowed for the investigation of myxobacterial pan-genomes, i.e. the complete set of genes found within members of a taxon. Genes are described as “core” if they are present in every member of a taxon, or “accessory” if only found in certain taxon members. Myxobacterial pan-genomes contain unusually large proportions of accessory genes. For instance, when comparing 10 strains within the *Myxococcus* genus, less than 10% of the genes in each strain were found to be core (90% accessory), and 80% of pan-genome genes were found to be present in only a single strain (21). Such extreme genetic individuality may relate to the myxobacterial predatory lifestyle. For example, does the accessory pan-genome act as a reservoir of genes with activity against specific prey? Many such questions remain regarding the factors that govern myxobacterial genomes, including the following: How quickly are accessory genes gained/lost? How is synteny maintained in the face of gene gain/loss? Does HGT favor the acquisition of genes from prey? What selective pressures act on genes of the accessory pan-genome, and how do they confer adaptive benefits? Answers to these questions will advance our understanding of myxobacterial genome evolution, shedding light on the mechanisms that drive genetic diversity, adaptation, and ecological interactions in their predatory multicellular lifestyle.

## *M. XANTHUS* AS THE FOUNDING BACTERIAL MODEL FOR MULTICELLULAR DEVELOPMENT

Contrary to the term “multicellularity” used in a loose sense for other bacteria (in which cells simply co-exist together in amorphous biofilms), in *M. xanthus,* it involves differentiation of cell fates, programmed cell death, and the formation of higher-order structures such as fruiting bodies, which are all hallmarks of “true” multicellularity as defined for organisms in the metazoan kingdom. This is one of the reasons why *M. xanthus* is such an exciting model organism; it is possible to study foundational “metazoan” principles in a tractable bacterial system. In fact, the development of fruiting bodies under nutrient-poor conditions is arguably the most striking feature of myxobacteria. The morphology of these macroscopic structures differs from one species to another, varying from simple mounds to sophisticated structures consisting of one stalk with several sporangioles (Fig. 1).

**Fig 2:**
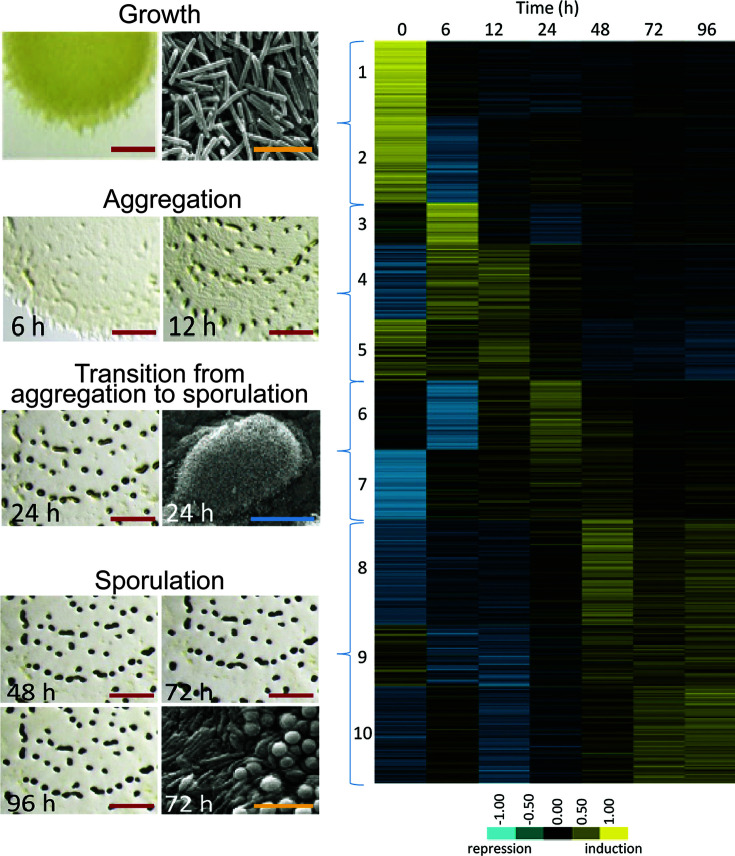
Relative expression profiles of significantly regulated genes at the indicated hours post-starvation induction. Genes were clustered into 10 developmental groups based on the time of peak expression and then organized according to the temporal progression of development. Developmental group number and the stages of the developmental program (with images of aggregates under a dissecting microscope and cells under a scanning electron microscope) are indicated on the left. In the images, red, blue, and yellow bars represent 2 mm, 100 µm, and 5 µm, respectively. Modified from Reference (30).

The Dworkin group was the first to show that fruiting-body formation requires high cell density, starvation, and motility on a solid surface (6, 27, 28). For *M. xanthus* encountering nutrient deficiencies, four distinct fates await cells in these swarms. Cells at the swarm periphery continue their net outward migration foraging for sustenance (*fate 1*), while those closer to the swarm interior participate in a developmental program that is completed in 72 h and is divided into two stages: aggregation and sporulation. During the first 24 h, rod-shaped vegetative cells aggregate and pile on top of one another to build macroscopic mounds. Within the mounds, sporulation begins, with ~10% of rods differentiating into myxospores (*fate 2)* (Fig. 2). For the remainder of cells, a distinct subset maintains their rod morphology (peripheral rods, akin to persister cells) and forms a base for the fruiting body (*fate 3*), while others undergo a cell-death program that leads to cell lysis (*fate 4*), releasing nutrients to complete development. When nutritional conditions are once again favorable, myxospores inside the fruiting bodies germinate, resuming a new vegetative cycle as a small cooperatively-feeding swarm (28, 29).

The advent of molecular biology enabled the establishment of the genetic basis of development and its control, and various mutants were generated that were defective in aggregation and/or sporulation (31). Using the Tn*5* transposon*,* Kuner and Kaiser devised a method where mutant phenotypes could be easily screened and linked to particular genes involved in development, motility, or other processes (32). Using extracellular complementation via mixing of two strains, conditional mutants were isolated with apparent defects in extracellular developmental signals (33). These and other studies revealed that *M. xanthus* cells communicate to coordinate behaviors during development, with five complementation groups or signals being identified. Two of these signals (A and C) have been extensively characterized (34–37).

The A-signal regulates the early stages of development and consists of a mixture of diffusible amino acids and peptides that function as a *M. xanthus*-specific quorum-sensing signal to monitor whether the cell density of the swarm is sufficient to ensure fruiting-body formation. By contrast, the C-signal is the product of the *csgA* gene and remains associated with the cell, being transmitted via cell–cell contact. Again showing the broad applicability of *M. xanthus* research, the CsgA protein not only functions as a signal that coordinates multicellular development (34, 38–40) but also has cardiolipin phospholipase activity that led to the discovery of *Drosophila* and human proteins with similar activity, which may explain their role in preventing neurodegenerative disorders (37). The C-signaling pathway functions throughout the developmental cycle and plays different roles depending on the protein level. At low levels, the C-signal induces rippling, that is, coordinated rhythmic movements associated with starvation-induced development of some strains. At medium levels, aggregation is induced, and at high levels inside aggregates, sporulation is induced, producing a population of myxospores ready to germinate together when nutrients become available once again.

## *M. XANTHUS* AS A MODEL GRAM-NEGATIVE BACTERIUM FOR SPORULATION

Sporulation is a strategy utilized by a wide variety of organisms to survive unfavorable conditions (41). Myxospores, initially called “microcysts”, range from phase-bright and spherical (e.g., *Myxococcus*) to opaque “short, fat rods” (e.g., *Sorangium*), but all are obviously morphologically distinct from the respective vegetative cells (42). Spores of *M. xanthus* are spherical (containing a thick spore coat), metabolically quiescent, and display increased resistance to heat, desiccation, UV irradiation, detergents, enzymatic digestion, and sonic disruption (43, 44).

Sporulation of myxobacteria commonly occurs inside fruiting-body structures. However, at least in *M. xanthus* and *Stigmatella aurantiaca*, spores can be directly and rapidly induced in nutrient-replete vegetative cells by the addition of high (non-physiological) concentrations of certain chemicals such as glycerol, ethylene glycol, propanol, phenethyl alcohol, and dimethyl sulfoxide in a process referred to as chemically induced sporulation (7, 10, 45, 46). Chemically induced myxospores exhibit resistance to environmental challenges, though to a lesser extent compared to those derived from fruiting bodies, likely because of their thinner protective coats. While chemical induction of myxospore formation can be uncoupled from starvation-induced sporulation during development (47), both spore types share a similar core sporulation genetic program (30, 48). They have thus been invaluable in characterizing the molecular mechanisms of both spore morphogenesis and germination (49, 50).

To date, most knowledge on bacterial spore germination is limited to studies on Gram-positive organisms (e.g., Bacilli and Clostridia), which form endospores via asymmetric division (51), while sporulation research on Gram-negative bacteria is more scarce. In *M. xanthus*, spore morphogenesis is fundamentally different in that cell division is not required. Instead, the entire ~7 μm × 1 µm rod-shaped vegetative cell transforms into a spherical spore, ~1.7 µm in diameter. Of particular interest is the massive rearrangement of the cell envelope; peptidoglycan (PG) in the periplasm is degraded, and a novel carbohydrate-rich spore coat sacculus is deposited on the surface of the outer membrane (OM). In addition, *M. xanthus* spore germination also involves social interactions (52).

Chemical induction of sporulation is immediately accompanied by a massive metabolic reorganization, including the upregulation of the gluconeogenesis and polysaccharide synthesis pathways, consistent with increased production of protective or storage compounds and the spore-coat polysaccharide (48, 53–55). Spore morphogenesis begins with shortening and widening of the rods, likely driven by MreB-dependent PG remodeling (48). It is presumed that the actin homolog MreB coordinates the degradation of existing PG, which is ultimately absent in the mature spore (49, 56). If cells are nutrient-limited prior to shape change, concurrent with cell shortening, excess inner membrane (IM) lipids are removed and deposited into cytoplasmic lipid bodies that are later used as an energy source to drive spore maturation (37, 57, 58). Finally, cells must also assemble the coat on the spore surface (outside of the OM).

## FRESH OPPORTUNITIES FOR PROBING PG DYNAMICS

Because PG is the major stress-bearing structure that determines cell shape (59, 60), the morphological transition during *M. xanthus* sporulation requires large-scale PG remodeling. PG is a widely conserved, bacterial-specific structure. The entire PG layer is one continuous, mesh-like macromolecule of glycan strands cross-linked via short peptides (61). PG precursors are synthesized in the cytoplasm and flipped across the cytoplasmic membrane (62, 63), where glycosyltransferases (GTases) polymerize them into glycan strands, which are then crosslinked into the PG network by transpeptidases (TPases). In most rod-shaped bacteria, including *M. xanthus*, two conserved polymerase systems assemble PG during cell elongation: the Rod system and class A penicillin-binding proteins (aPBPs) (62, 64, 65). Besides polymerases, the growth and modification of covalently closed PG also require hydrolases to generate openings in the existing PG network (62, 65, 66).

aPBPs possess both GTase and TPase activities and thus were considered the major PG synthases. By contrast, class B penicillin-binding proteins (bPBPs), which only contain TPase domains, were considered secondary. Seminal works from the Bernhardt group changed this view in 2016. They showed that bPBPs, together with their partner GTases and specific cytoskeletons, form the PG synthase complexes for PG elongation and division (64, 67–69). The GTase RodA and TPase PBP2, orchestrated by MreB, form the core of the Rod system, which determines rod shape and carries out most of the PG expansion during vegetative growth (64, 69–73). By contrast, aPBPs do not depend on MreB, and the absence of single aPBPs rarely abolishes cell survival or rod-like morphology (74–76). Dethroned from the major PG builders, the physiological functions of aPBPs must be re-examined (77, 78).

*M. xanthus* expresses a conserved set of PG polymerases, including three aPBPs, a Rod system, and 35 putative PG hydrolases (20). As every PG-related enzyme in *M. xanthus* has homologs in *E. coli*, in the early years, *M. xanthus* did not show significant potential as a model organism for studying PG dynamics. This perception changed in 2009, when Bui et al. discovered that chemically induced *M. xanthus* spores do not contain PG (56). This surprising finding has two implications. First, because rod-shaped *M. xanthus* cells thoroughly dismantle their PG to transform into spherical spores, the sporulation process provides an invaluable opportunity to study PG degradation. Second, when *M. xanthus* spores germinate, cells must rebuild a rod-shaped PG sacculus without preexisting templates, which is a unique system for studying PG synthesis and rod-like morphogenesis (49, 50). Work on *M. xanthus* sporulation and germination has revealed new insights into bacterial PG dynamics, which would not have been attainable through the study of other model bacteria.

DacB (homolog of *E. coli* PBP4) is the first hydrolase identified for PG degradation during the rod-to-sphere transition in sporulating cells. Importantly, DacB also causes PG damage in vegetative cells under the stress of moenomycin, an antibiotic that specifically inhibits aPBPs (79). Using single-particle microscopy, Zhang et al. discovered that when PBP1a2 (one of the three aPBPs) binds to moenomycin, it specifically promotes binding and degradation of PG by DacB. This finding not only clarified the action of moenomycin but also revealed the first example of functional coordination between PG synthases and hydrolases. As bacterial cells usually accumulate hydrolase-cleaved PG fragments when treated with the antibiotics that inhibit PG polymerases (80–82), such coordination could be conserved in many bacteria.

Although aPBPs and the Rod system both contribute to PG growth, how their labor is divided remains unclear. During germination, *M. xanthus* spores first grow spherical PG, in which both aPBPs and the Rod system participate in PG assembly. By contrast, once the germinating spores start to elongate into rods, the Rod system becomes the dominant machinery for PG growth (49). During the sphere-to-rod transition, the MreB cytoskeleton connects the Rod machinery to the Ras-like GTPase MglA (see details below), which breaks symmetry and establishes cell poles (49, 83–85). As MreB is an ancient module that determines rod shape (86), and Ras-like GTPases are conserved in both prokaryotes and eukaryotes (87), these findings suggest that the interplay between GTPase, cytoskeletons, and cell wall-related enzymes might be a conserved mechanism for cell polarization that has evolved before the divergence between prokaryotes and eukaryotes.

## DEVELOPMENTAL GENE EXPRESSION

Driving the morphological changes during fruiting -body formation is a complex signaling and gene regulatory network. Kroos et al*.* performed the first genome-wide hunt for developmentally regulated *M. xanthus* genes and identified ~30 developmental markers using Tn*5 lac* as a discovery tool (31). Since then, many regulators have been found to modulate the expression of genes at different stages of development and are classified into three sequentially-acting modules (88). The first module, consisting of a cascade of enhancer-binding proteins (EBPs), activates the expression of early genes (89–92). The second Mrp module depends on the previous one and is responsible for the accumulation of MrpC (93, 94). The third FruA module depends on the Mrp module (93) and, together with MrpC, regulates the expression of many genes important for sporulation (88, 95). The EBP cascade and the MrpC/FruA cascade are interconnected cascades of signal-responsive transcription factors that regulate well over 1000 genes.

Genome sequencing of myxobacteria allowed us to better understand their genetic potential and perform transcriptomic studies to elucidate the transcriptional changes that cells undergo during their complex lifecycles. Hundreds of upregulated and downregulated genes were discovered using DNA microarrays (96–98), and comparison of these *M. xanthus* genes with those in other myxobacteria revealed unexpected plasticity in their developmental programs (98). The first RNA-seq study spanning the entire time course of starvation-induced development found that ~20% of the genes encoded in the *M. xanthus* genome are differentially regulated at specific time points (30) (Fig. 2). Analysis of developmentally regulated genes has thus led to numerous conclusions. First, the two motility systems (described below) exhibit different expression profiles. Second, glycogen and lipids are consumed to yield sufficient energy to complete fruiting-body formation under nutrient deprivation. Third, amino acid and sugar precursors needed for specific developmental macromolecular synthesis appear to be released by protein and polysaccharide turnover and gluconeogenesis. Fourth, several secondary metabolites are produced in larger amounts during development. Fifth, translation may be rewired.

In addition, 77 transcriptional regulators (including one- and two-component systems, and sigma factors) plus 22 serine/threonine protein kinases are developmentally regulated and are expected to regulate differential gene expression during development. Recently, Cappable-seq provided a fantastic resource for studying developmental gene expression by mapping transcriptional start sites during the first 24 h post-starvation (99). Using this technique, the promoter regions of several late-acting developmental operons were identified (100). The results suggest that each operon is regulated uniquely, under eukaryote-like combinatorial control of two or three transcription factors. However, more studies are needed to understand all the mechanisms employed by these social bacteria to build multicellular fruiting bodies.

## SIGNALING ROLES OF NUCLEOTIDE DERIVATIVES IN MYXOBACTERIA

Along with the transcriptional regulation network, a variety of nucleotide derivatives also play critical roles in *M. xanthus* development. Their importance was first revealed in the 1970s, with cyclic adenosine monophosphate (cAMP) and cyclic guanosine monophosphate (cGMP) found to play different roles. The Zusman group identified cAMP as an important inducer of *M. xanthus* fruiting-body formation (101, 102), while the McCurdy laboratory demonstrated cGMP to be a crucial aggregation attractant (103, 104). During development, levels of cAMP and cGMP were found to peak at different times, preceded by spikes in the activity of the relevant cyclases (105). While *E. coli* contains a single AMP cyclase and a single cAMP-binding protein, *M. xanthus* encodes at least 10 putative AMP/GMP cyclases and numerous potential cAMP/cGMP-binding proteins (19), though only one of the latter has been experimentally identified thus far (106). The putative cAMP-binding protein MrpC is a major early regulator of development (107). Interestingly, MrpC was recently found to directly repress the synthesis and activate the degradation of another second messenger, cyclic di-GMP (c-di-GMP), during development (99).

c-di-GMP has emerged as a ubiquitous and highly versatile signaling molecule that regulates the transitions between motility and sessility in bacteria (108). In *M. xanthus*, c-di-GMP regulates multicellular development and cellular differentiation; a minimum threshold of c-di-GMP is essential for fruiting-body formation and sporulation (109). Interestingly, while *M. xanthus* encodes 19 putative c-di-GMP synthetases, only one appears to be essential for development (109, 110). c-di-GMP also regulates *M. xanthus* type IV pili (T4P)-dependent motility, exopolysaccharide (EPS) production, cell polarity, and even DNA organization and segregation, indicating that *M. xanthus* is an ideal model organism for studying the versatility of c-di-GMP (111–114). Recently, the Hammond group discovered that *M. xanthus* produces a novel cyclic dinucleotide, cyclic AMP-GMP (cGAMP), that is specific to its surface-dwelling lifestyle (115). Further analysis revealed that cGAMP could promote resistance against osmotic stress through unknown mechanisms (116). In addition, cGAMP production is activated by cAMP but inhibited by c-di-GMP, suggesting complex cross-regulation among nucleotide derivatives (115, 116).

(p)ppGpp is the main mediator of the “stringent” response in bacteria, which connects protein synthesis (ribosome function) to amino acid availability through the ribosome-associated protein RelA (117). Besides the stringent response, (p)ppGpp in *M. xanthus* and *S. aurantiaca* is responsible for “sensing” starvation and the initiation of the developmental program (118, 119). Since both of these myxobacterial species primarily consume proteins, fats, and alpha-keto acids as their primary carbon source, it was hypothesized that the stringent response is used by cells to monitor their nutritional status via their translational capacity (118, 119).

## *M. XANTHUS* POLYSACCHARIDES: PIVOTAL PLAYERS IN PHENOTYPIC PLASTICITY AND PHYSIOLOGY

**Fig 3:**
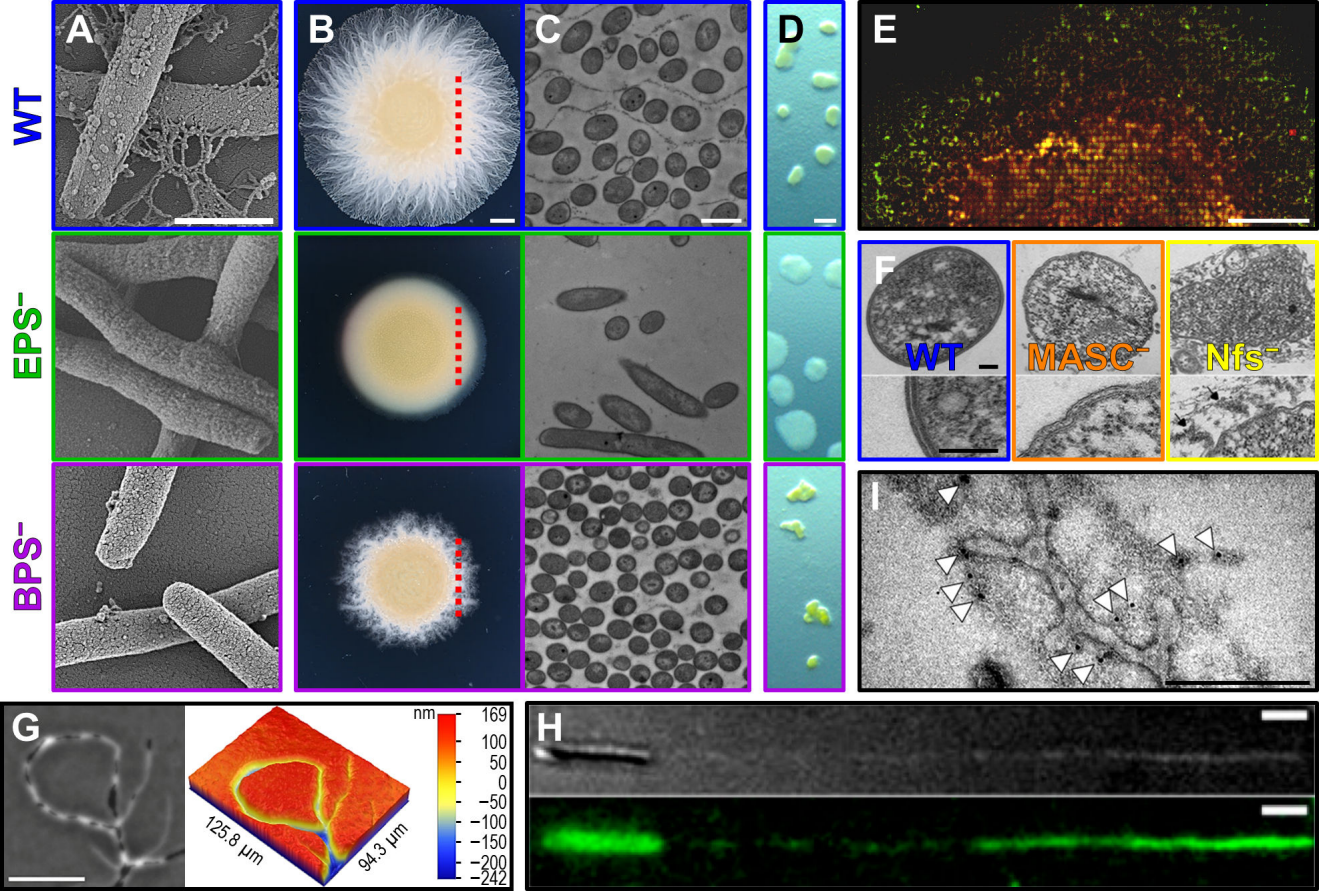
Polysaccharide-mediated *M. xanthus* physiology. (**A**) Scanning electron microscopy of WT, EPS^−^, and BPS^−^ *M. xanthus* cell surfaces (scale bar: 1 µm), adopted from Reference (120) with permission. (**B**) T4P-dependent motility of WT, EPS^−^, and BPS^−^ *M. xanthus* cells (scale bar: 2 mm). *Red dashed line:* relative location of slice presented in Panel C. (C) Transmission electron microscopy cross-section of *M. xanthus* polysaccharide mutant swarms (scale bar: 1 µm), adopted from Reference (120) with permission. (**D**) Fruiting-body formation of WT, EPS^−^, and BPS^−^ *M. xanthus* cells (scale bar: 400 µm), adopted from Reference (55). (**E**) Tiling fluorescence microscopy of swarming cells expressing EPS and BPS machinery linked to green and red fluorescent reporters, respectively (scale bar: 400 µm), adopted from Reference (55). (**F**) Transmission electron microscopy of myxospores from WT, MASC^−^, and Nfs-mutant cells (scale bar: 0.1 µm), adopted from Reference (128). (**G**) Phase-contrast microscopy (*left side*, scale bar: 50 µm) and 3D optical profilometry (*right side*) of *M. xanthus* trails left behind at the agar-air interface, adopted from Reference (129). (**H**) Wet-SEEC high-refraction microscopy (*top*) and fluorescence microscopy (*bottom*) of *M. xanthus* cells (treated with fluorescent ConA lectin) gliding on a chitosan-functionalized glass substratum in a PDMS microfluidic device (scale bar: 1 µm), adopted from Reference (130). (**I**) Transmission electron microscopy of a negatively stained “slime” trail left behind a *M. xanthus* cell gliding across a chitosan-functionalized copper electron microscopy grid (scale bar: 250 nm). Lipid vesicles and lipid tubes are present in the electron-dense trail material. White arrows indicate colloidal gold particles associated with biotinylated ConA lectin, adopted from Reference (131).

In addition to gene regulation and signaling molecules, secreted and cell-surface polysaccharides impact every stage of the complex life cycles of myxobacteria. Vegetative *M. xanthus* cells secrete EPS and biosurfactant polysaccharide (BPS) (55), while sporulating cells secrete major spore coat polysaccharide (MASC) (54). EPS remains largely surface-associated and is the main constituent of the *M. xanthus* glycocalyx, playing a critical role in motility, biofilm structure, and stress protection (120–122). Early electron microscopy of *M. xanthus* led to the visualization of EPS projections thought to be appendages (originally termed “fibrils”) emanating from the cell surface (Fig. 3A) (123). In a reported first for bacteria, EPS also serves as a retraction signal for T4P; that is, the T4P of a given cell can extend and bind to the EPS glycocalyx of an adjacent cell, triggering T4P retraction, thus mediating group motility (Fig. 3B) and community organization during vegetative (Fig. 3C) and developmental phases (Fig. 3D) (124, 125). Interestingly, T4P extension, but not retraction, is required for EPS production (126, 127).

BPS is secreted into the extracellular milieu and does not remain cell-associated (55). Unlike some bacterial biosurfactants, BPS does not act as a substratum-wetting agent secreted at the swarm front. Instead, it disrupts the integrity of the cell-surface EPS glycocalyx (resulting in EPS “fibrils”) (Fig. 3A), impacting single-cell motility, swarm-level spreading (Fig. 3B), and architecture (Fig. 3C), as well as fruiting-body formation (120, 122) (Fig. 3D). Contrary to EPS, BPS production is higher in cells that cannot build T4P. Within spreading swarms, BPS and EPS production is spatially modulated, with the respective machineries more highly expressed in the swarm interior and at the periphery, respectively (55) (Fig. 3E). Both BPS and EPS act as “shared goods” that restore normal behavior in deficient cells when provided exogenously (55, 120, 132).

MASC is only produced during development (55, 120). It blankets myxospores (Fig. 3F), making the latter phase-bright (48, 128, 133, 134). The exact manner by which MASC is packaged to form the rigid spore-coat sacculus is not known, but the process functions akin to winding loose yarn strands around a ball; secreted MASC becomes tightly wound around the myxospore surface via directed transport of the Nfs trans-envelope protein complex (paralogous to the gliding motility machinery, described below) by the AglR/Q/S proton-powered motor (48, 128). Following secretion, processing by the Nfs complex also results in shorter MASC fragments and may involve cross-linking with a glycine-rich peptide bridge (53).

EPS, BPS, and MASC are synthesized via separate Wzx/Wzy-dependent pathways (55), where lipid-linked sugar repeats are flipped from the cytoplasmic to the periplasmic leaflets of the IM and polymerized to preferred lengths (135). OM export of polymers from such pathways (136) was believed to be universally mediated by outer-membrane polysaccharide export (OPX)-protein octamers that partially traverse the periplasm and span the OM via an α-helical pore (137). However, the *M. xanthus* EPS-, BPS-, and MASC-pathway OPX proteins lack OM-spanning domains (138, 139), leading to the paradigm-shifting revelation that OPX proteins encoded throughout the bacterial kingdom fall into three structural classes, with the majority (including those in Gram-positive species with no OM) only containing “periplasmic” domains (138). In Gram-negative bacteria, such “truncated” OPX proteins instead function together with partner OM β-barrel porins to mediate polysaccharide export (138, 139).

As a diderm Gram-negative bacterium, the surface leaflet of the *M. xanthus* OM is constituted of lipopolysaccharide (LPS), but with uncommon properties (140, 141) such as branched lipid-A fatty acids, and only one Kdo (3-deoxy-D-*manno*-oct-2-ulosonic) sugar (itself modified with phosphoethanolamine) in the core oligosaccharide domain (142, 143). The O-antigen, while conventional in structure, impacts motility, swarm expansion, and fruiting-body formation (144). It remains to be seen whether these properties are important for the dynamic nature of the *M. xanthus* OM (described below).

During early research by Jahn (1924), microscopy revealed phase-bright trails left by single motile myxobacteria on agar (Fig. 3G, *left*), which became widely attributed to “slime” secretion at the lagging cell pole (145, 146). Cells were also found to follow these trails (147), leading to the “slime-trail” deposition-and-following hypothesis long associated with *M. xanthus*. However, recent 3D optical profilometry (129) revealed these phase-bright trails are actually furrows carved into the agar, appearing bright in phase-contrast microscopy, a notion originally proposed over 70 years ago by Meyer-Pietschmann (148) (Fig. 3G, *right*). Trail-following by *M. xanthus* on agar is thus an example of sematectonic stigmergy, i.e. physical modifications by leader cells of the surrounding environment guide followers without direct communication (129). Nevertheless, *M. xanthus* cells lacking O-antigen polysaccharide or deficient in EPS, BPS, and MASC combined still deposit carbohydrate-rich trails on non-deformable glass. Trail regions with more material correlated with slower gliding, suggesting adhesive rather than lubricative properties (130) (Fig. 3H). Electron microscopy revealed that trails contained polysaccharides along with debris from OM vesicles and tubes (Fig. 3I), indicating their compositional heterogeneity (131). Whether these findings point to the presence of a novel secreted *M. xanthus* polysaccharide remains unknown. Overall, many mysteries remain as to the production and function of polysaccharides by this intriguing bacterium, including their involvement in *M. xanthus* motility.

## SOCIAL MOTILITY AND TYPE IV PILI

*M. xanthus* lacks flagella and thus its movement across surfaces was historically referred to with the catch-all term of “gliding motility,” previously used to describe any form of smooth surface motility along the long axis of cells and not involving observable appendages. However, in a string of foundational papers, Hodgkin and Kaiser isolated motility mutants and demonstrated that *M. xanthus* has two genetically distinct motility systems, commonly referred to as the A (adventurous)- and S (social)-motility systems responsible for single-cell and cell-group movement (respectively) away from the swarm edge (149–151).

Polarly localized T4Ps were first implicated in myxobacterial motility by Howard McCurdy and colleagues (152–154). In 1995, Wu and Kaiser provided the definitive evidence that T4Ps are required for S-motility via the identification and disruption of T4P genes (155). As such, the “social” type of *M. xanthus* motility is in fact T4P-dependent swarm spreading.

**Fig 4:**
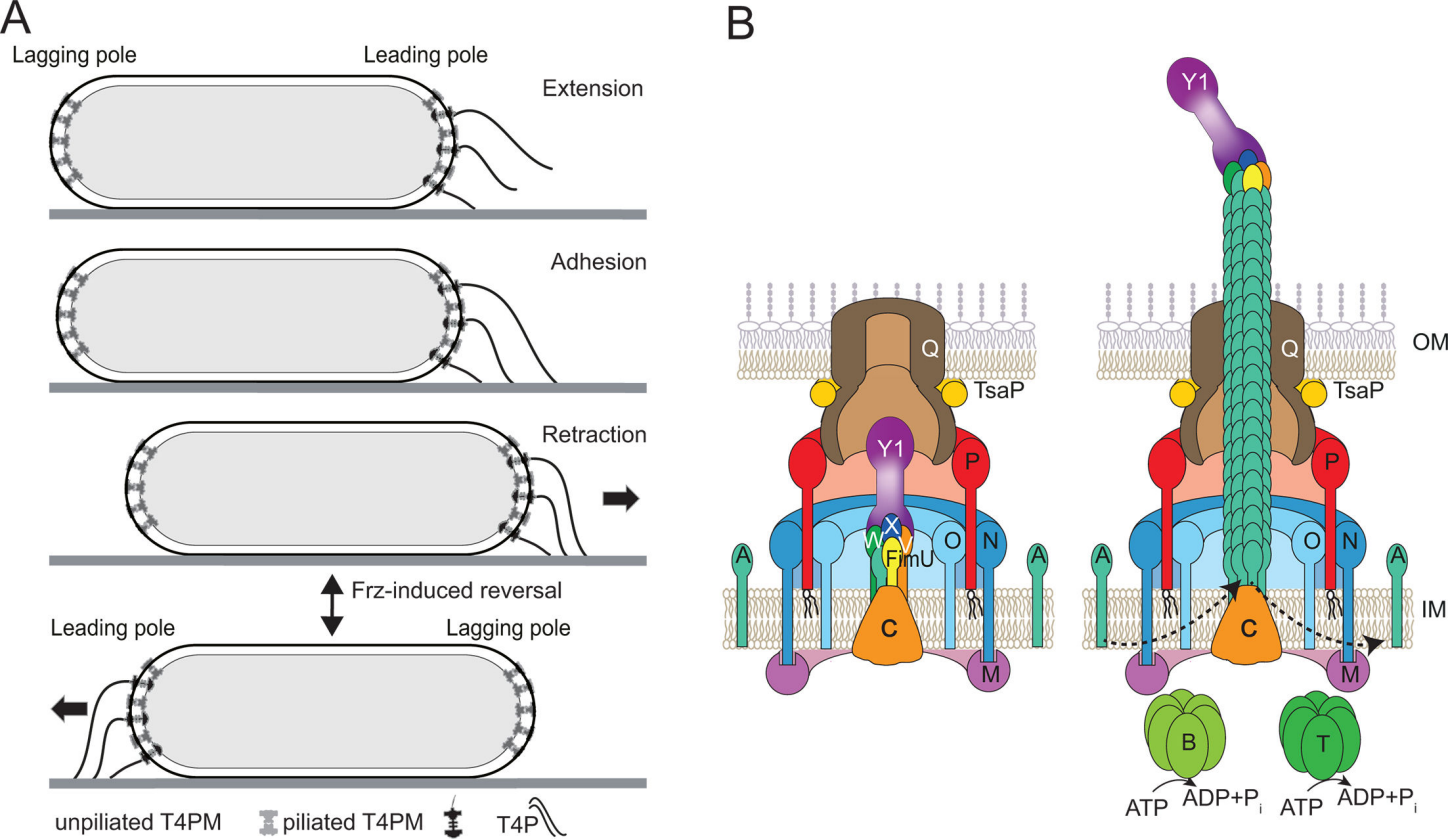
T4P-dependent motility and the T4PM. (**A**) T4P-dependent motility depends on dynamic cycles of extension, adhesion, and retraction. The T4PM is present at both cell poles, but only active at the leading pole. During reversal, the leading/lagging polarity switches, enabling the activation of the T4PM at the new leading pole. (**B**) The architecture of the T4PM of *M. xanthus* in two states. Left, unpiliated state. The core T4PM consists of four functional elements, that is, an OM pore (PilQ and TsaP), an alignment complex (PilP, PilN, and PilO) that connects the OM pore to the IM platform complex (PilC and PilM), and a priming complex (four minor pilins (orange, yellow, blue, and green) and PilY1). Right, piliated T4PM with an extended T4P capped by the tip complex. PilB and PilT associate with PilM and PilC in a mutually exclusive manner for extension and retraction, respectively. Dashed arrows indicate incorporation at, and removal from, the pilus base of PilA during extension and retraction, respectively. Proteins labeled with single letters have the Pil prefix.

T4Ps are highly dynamic structures that undergo cycles of extension, surface adhesion, and retraction (156). Because T4Ps adhere strongly to surfaces, their retraction pulls cells forward. In fact, structural and biophysical analyses have demonstrated that the *M. xanthus* T4P is the strongest, most compact, and most rigid T4P yet described (157, 158). In rod-shaped *M. xanthus* cells, the machine that drives the T4P extension/retraction cycles is present at both cell poles, but is only active at the leading pole, thereby ensuring unidirectional translocation (Fig. 4A) (159–162). This unique localization pattern of the (in)active T4P machine (T4PM), together with the tractability of *M. xanthus* for cryo-electron tomography (163), made it the model of choice to determine the overall T4PM architecture in bacteria (164, 165).

The T4PM is composed of 15 different proteins (156), most of which are present in multiple copies that localize to the OM, periplasm, IM, and cytoplasm. Thirteen of these proteins form a structure that spans the entire cell envelope, constituting the unpiliated T4PM at both cell poles (Fig. 4B, *left*). In the piliated T4PM at the leading cell pole, the two remaining proteins, PilB (extension ATPase) and PilT (retraction ATPase), both forming hexamers, come into play. PilB binds to the cytoplasmic base of the T4PM (166) (Fig. 4B, right). Upon ATP hydrolysis, PilB has been suggested to rotate PilC (164, 167), resulting in the addition of the major pilin subunit PilA to the base of the growing pilus. Initially, PilA subunits are added to the minor pilin/PilY1 priming complex, and therefore, this priming complex ultimately localizes to the pilus tip, where it is predicted to be involved in adhesion (165) (Fig. 4B, right). While PilB stimulates T4P extension, PilT is essential for retractions (126, 156, 168). The swap from PilB to PilT, and thus initiation of retraction, is either predicted to be a stochastic event or induced by adhesion of the T4P tip to the surface (164, 169). In the latter model, tip adhesion would induce conformational changes in the pilus that are communicated to the base of the T4PM, causing the release of PilB. Upon PilB release, PilT binds to the T4PM, hydrolyzes ATP, and rotates PilC in the opposite direction, thereby removing PilA subunits from the pilus base (164, 167). The return of the minor pilin/PilY1 complex into the T4PM stops retraction. PilT is then released, and PilB can bind for a new round of T4P extension (165).

In contrast to other model organisms such as *Pseudomonas* and *Neisseria* species, *M. xanthus* has the potential to form three different minor pilin/PilY1 priming complexes (165, 170, 171). As a consequence, *M. xanthus* T4Ps can potentially have three different tip complexes, which may recognize distinct targets, thereby enabling adhesion and T4P-dependent motility across different surfaces (165, 171).

## GLIDING MOTILITY AND THE FLUID GLIDING MACHINERY

Nowadays, “gliding” (i.e., A-motility) in reference to *M. xanthus* motility denotes the smooth movement of single cells on a substratum, independent of T4P function (172–174). Chemical and UV mutagenesis were first used by MacRae and McCurdy to generate non-gliding *M. xanthus* strains (153), then by Hodgkin and Kaiser to isolate mutants that were no longer singly motile but could still move in groups (150, 151). Later transposon-based screens in a parent strain that could not move by S-motility identified 35 potential genes required for gliding (175). However, these discoveries did not elucidate the gliding mechanism.

**Fig 5:**
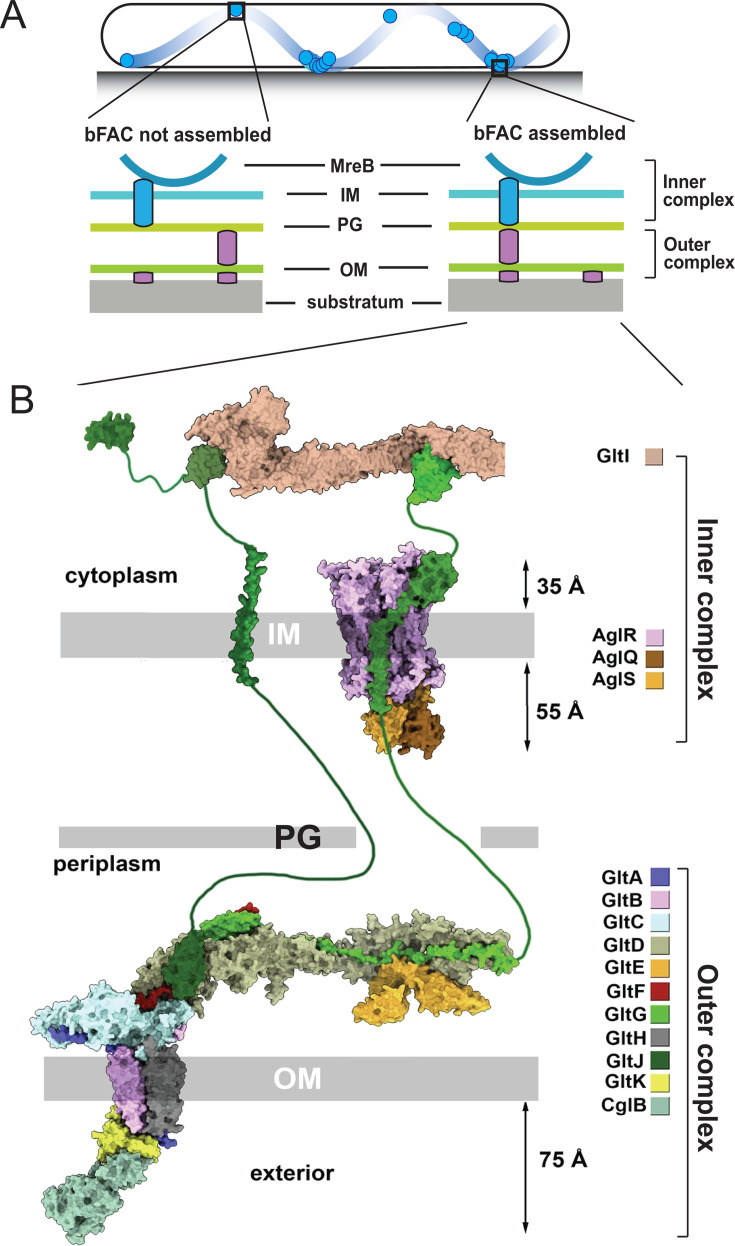
Gliding motility and the bFAC. (**A**) Stationary bFACs (relative to the substratum) drive *M. xanthus* gliding motility. Motors carrying incomplete gliding complexes either diffuse or move rapidly along helical paths but do not generate propulsion. Motors stall and become nearly static relative to the substrate when they assemble into complete bFACs with other motor-associated proteins at the ventral side of the cell. Stalled motors push MreB and bFACs in opposite directions and thus exert force against OM adhesins. Overall, as motors transport bFACs toward lagging cell poles, cells move forward, but bFACs remain static relative to the substratum. Reproduced from Reference (183). (**B**) Structural model of the bFAC predicted by AlphaFold (184), modified from Reference (185). Note that whether and how GltG and GltI thread through PG are not investigated by experiments. IM, inner membrane; OM, outer membrane; PG, peptidoglycan.

In 2007, fluorescently labeled AglZ (a cytoplasmic protein involved in gliding) was shown by Mignot et al. in gliding cells to form bright clusters at the leading pole and fainter, nearly evenly spaced clusters along the cell body. These AglZ clusters remained fixed in space relative to the substratum as cells advanced and were hence being transported toward the lagging cell pole at the speed of the cell. Over time, new clusters were found to assemble at the leading pole while old clusters were dispersed at the lagging pole. With similarities to focal adhesion complexes in migrating eukaryotic cells, these observations in *M. xanthus* suggested that there were yet-to-be-identified gliding motors in the bacterium that could exert force on several bacterial focal adhesion complexes (bFACs), resulting in forward locomotion (176) (Fig. 5A). This energy-harvesting machinery was later identified as IM AglR/Q/S, possessing significant homology to MotA/B of the *E. coli* flagellar stator, which forms proton channels that convert the proton-motive force into mechanical force (177, 178). Periplasmic AgmU (GltD), another essential protein for gliding motility, was later found to also localize to bFACs. Using pull-down experiments, a gliding transducer apparatus spanning the cytoplasm, IM, and periplasm was then discovered (179). OM gliding transducer components were subsequently identified (180, 181), with five of these (including OM lipoprotein and integral-OM β-barrel constituents) found to co-purify with the CglB cell-surface lipoprotein adhesin (182). Together, these findings paint the picture of an envelope-spanning bFAC consisting of ~20 different protein components that associate with each other in an interdependent manner (Fig. 5B).

Strikingly different from other motility systems, such as flagella and T4Ps, *M. xanthus* bFACs appear to lack a rigid structure that traverses the entire cell envelope. Using single-particle microscopy, Nan et al. reported that the AglR/Q/S motors are not fixed in the cell envelope but rather travel the length of the cell along helical trajectories. The motors stall in bFACs when they travel to the sites where the cell contacts the substratum (Fig. 5A) (85, 177, 186). In 2016, Faure et al. clarified that only the motors that stall in bFACs produce force for gliding, while the ones that move along helical trajectories carry incomplete gliding complexes (lacking OM components), which do not generate propulsion (187). At sites of cell–substratum contact, Islam et al. then revealed the cytoplasmic–IM–periplasmic gliding assembly engages the OM gliding complex, resulting in unmasking of the surface lipoprotein adhesin CglB (loaded in the OM assembly) and its binding to the substratum. This provided the first explanation by which forces generated by IM-embedded motors could be transduced across the cell envelope to the substratum (182) (Fig. 5B).

Ultimately, to arrive at a consensus model for the gliding mechanism, the so-called “PG problem” still had to be resolved, namely, how does the rigid PG layer impact transmission of mechanical force from the IM to the cell surface by fluid bFACs? As motors reside in the fluid IM, to transmit force to the cell surface, bFACs must push against two relatively rigid structures, one on each side of the IM, in opposite directions (188) (Fig. 5B). MreB filaments in the cytoplasm and PG in the periplasm likely provide such rigid support. While bFACs have been known for over 10 years to connect to MreB filaments (84, 85, 177, 189, 190), a connection between bFACs and PG was discovered only recently. While studying PG degradation, Ramírez Carbó et al. found that AgmT, a PG lytic transglycosylase, is required for *M. xanthus* gliding. Different from other gliding-related proteins, AgmT itself does not assemble into bFACs. Using single-particle tracking microscopy and coprecipitation assays, AgmT was found to be essential for the bFACs to connect to PG. Surprisingly, heterologous expression of *E. coli* MltG rescued the connection between PG and bFACs, restoring gliding motility. Hence, the lytic transglycosylase activity of AgmT anchors bFACs to PG, potentially by modifying PG structure (183).

Unbeknownst to researchers in 1977, the importance of OM-module proteins for gliding also provided the first evidence for a process called “OM exchange” (OME)—a phenomenon thus far only characterized in myxobacteria—involving physical exchange of OM lipids and proteins between compatible strains (described below). Using chemical & UV mutagenesis, five classes of *M. xanthus* “conditional gliding” (*cgl*) mutants were identified that were incapable of single-cell gliding on their own; however, pairwise mixing of cells between these five mutant classes resulted in transient restoration (termed “stimulation”) of single-cell gliding to the overall population, suggesting that the different respective missing gliding components had been reciprocally transferred between the two initially non-motile classes of cells (149). The bulk transfer of OM gliding-module lipoprotein CglB from donor cells to non-gliding recipient cells was later shown to transiently restore single-cell gliding (similar to the transfer of OM lipoprotein Tgl and its restoration of T4P-dependent motility). This suggested that donor and recipient strains could temporarily fuse their OMs to allow for the transfer of OM material, including the missing motility components (191), a process finally imaged in real time at the single-cell level over 35 years after the first report of “stimulation” (131).

## FOLLOWING A CHEMICAL COMPASS: CHEMOTAXIS FOR SURFACE MOVEMENTS

To navigate their natural habitats, bacteria adjust their movements toward favorable conditions and away from toxic environments through a process known as “chemotaxis.” Hints of a chemotactic response in *M. xanthus* were first reported in 1962, when fresh cells, separated from a plate of aggregated cells by a semipermeable membrane, formed fruiting bodies directly above the aggregates (192). Direct evidence of chemotaxis was later provided in the 1970s using gradients in agar of either nutrients or messenger molecules cGMP or adenosine monophosphate (AMP), atop which swarms were found to expand more quickly up the concentration gradient rather than down it (103, 193).

**Fig 6:**
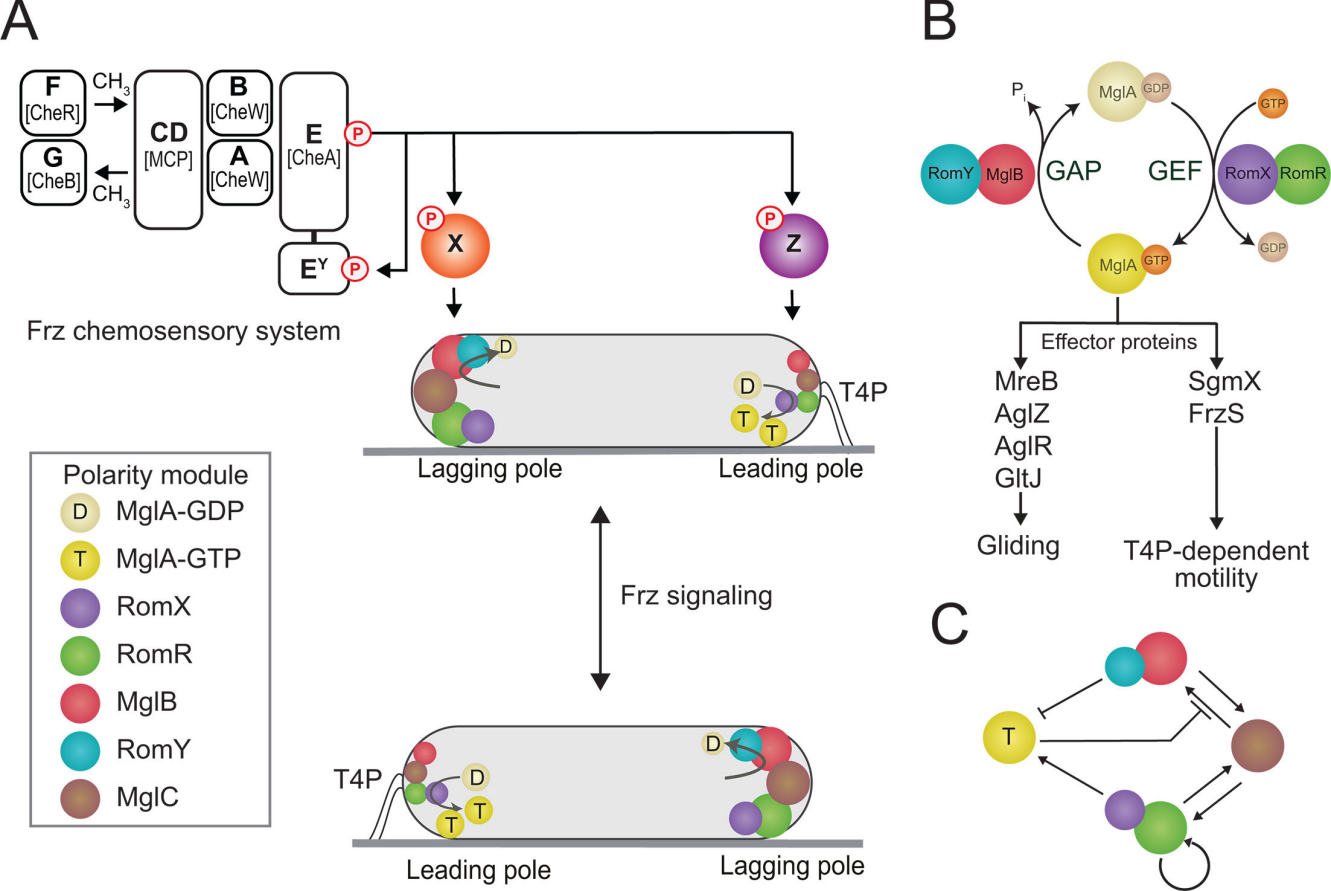
Polarity control of *M. xanthus* motility systems. (**A**) The polarity module and the Frz chemosensory system regulate motility. All proteins in the Frz system have the Frz prefix. Homologs of individual proteins in the *E. coli* Che system are indicated in brackets. Phosphate flow from the FrzE kinase is indicated by arrows. Phosphorylated FrzZ and FrzX localize at the leading and lagging poles, respectively, and are indicated to act on the polarity module. The cell below schematically illustrates the polar localization of the proteins of the polarity module with T4P at the leading pole. The circle sizes indicate the relative amount of a protein at a pole. (B) MglA GTPase cycle and interaction with downstream effectors to stimulate gliding motility and T4P-dependent motility. GEF, guanine nucleotide exchange factor; GAP, GTPase-activating protein. (**C**) Interactions between the proteins of the polarity module establish their asymmetric polar localization at the two poles, resulting in correct leading–lagging cell polarity. Colors as in A.

To move efficiently as large cell swarms or single cells, form fruiting bodies, and prey on other microorganisms, *M. xanthus* uses the Frz system (a homolog of the Che pathway in enteric bacteria) to regulate the frequency at which both the gliding- and T4P-dependent motility systems switch directionality within cells. After isolating nearly 2,000 mutants with defects in aggregation and/or sporulation, the Zusman group observed that certain mutants formed “frizzy” aggregates of multicellular filaments instead of well-defined spore-containing mounds. These mutations were all mapped to the same genetic locus (later termed *frz*) encoding Che-like proteins (194, 195). Central components of the Frz pathway appear divergent from their enteric counterparts: (i) FrzCD is a cytoplasmic methyl-accepting chemotaxis protein (MCP) that lacks an obvious sensing domain but instead possesses a DNA-binding domain that allows the formation of Frz clusters at the bacterial nucleoid (196, 197). While it is known that Frz proteins form clusters to respond to signals in a cooperative manner (as in other bacteria), the role of their association with the nucleoid is less clear. One hypothesis is that multiple clusters on the nucleoid could ensure that each daughter cell inherits sufficient Frz clusters and can thus have the capacity to reverse soon after cell division (198, 199). (ii) FrzE is a hybrid protein composed of a histidine kinase (CheA) and a response regulator (CheY) domain (200). FrzE can transfer phosphoryl groups to three CheY domains, (iii) one represented by FrzX and (iv) the other two within the di-CheY protein FrzZ (201) (Fig. 6A).

The identification of chemotaxis signals has been particularly challenging due to the lack of an obvious sensing domain in FrzCD. Some short-chain alcohols, like isoamyl alcohol (IAA), act as repellents as they increase the reversal frequency (202). Conversely, certain lipids act as attractants by decreasing the reversal rate (203, 204). Enteric bacteria adapt their chemotactic responses to given attractant concentrations, a behavior that allows them to reset the system and prepare a response to eventually reach higher concentrations. In *M. xanthus*, the apparent lack of adaptation to repellents raises questions about the precise mechanisms underlying chemotaxis-like behaviors in this bacterium (205). However, given that methylation and demethylation of FrzCD occur (206, 207), perhaps a suitable repellent to evoke adaptation has not yet been found. While *M. xanthus* cells can respond to certain linear gradients (103, 193), the bacterium may also respond to non-linear gradients of large molecules, prey, or even to mechanical cues (e.g., during rippling) (208).

In addition to the Frz system, *M. xanthus* encodes seven other chemosensory systems that regulate various other functions, illustrating how this bacterium has evolved to finely tune Che signaling pathways to modulate behaviors beyond taxis. For example, the Dif chemosensory system regulates the synthesis and export of EPS (204). Some of these Che-like systems might interact, transducing multiple signals into coordinated cellular responses, effectively acting as a bacterial sensory/signaling network or “brain.” For instance, the Che4, Che5, and Che6 systems interact to form a large chemosensory module (209). With its two motility systems, complex array of chemosensory modules, and the use of chemotaxis responses to perform sophisticated bacterial social behaviors, *M. xanthus* stands as a unique model for studying Che pathways—an area that still requires significant exploration for deeper understanding.

## SPATIOTEMPORAL REGULATION OF *M. XANTHUS* MOTILITY

Crucial for directional chemotactic movements, rod-shaped *M. xanthus* cells exhibit well-defined leading and lagging cell poles when moving across surfaces using gliding or T4P-dependent motility. This is made possible because the two motility machineries are highly polarized and only assemble at the leading cell pole. However, during a reversal of direction, the old lagging pole becomes the new leading pole, with both motility machineries assembling at the new leading pole (84, 159, 176, 210–212) (Fig. 6A). Thus, *M. xanthus* cells are faced with two regulatory problems: (i) they must ensure that both motility machines assemble at the same pole and that (ii) both motility machines switch polarity in unison during reversals. Two interfacing protein modules, the polarity module and the Frz chemosensory system, control motility in *M. xanthus* by dynamically regulating leading–lagging cell polarity. The polarity module establishes and maintains leading–lagging cell polarity, while Frz signaling induces an inversion of this polarity and, thus, reversals occur. Jointly, these two modules create a spatial toggle switch.

The small GTPase MglA is the key protein of the polarity module, and in its active GTP-bound state, localizes to and defines the leading cell pole (213, 214) (Fig. 6A). This localization is brought about by the remaining five proteins of the polarity module (Fig. 6A). In particular, the RomR/RomX complex has guanine nucleotide exchange factor (GEF) activity and stimulates the formation of the active GTP-bound state of MglA (215), while the MglB/RomY complex is a GTPase activating protein (GAP), which stimulates the conversion to the inactive GDP-bound state of MglA (213, 214, 216) (Fig. 6B). These two complexes, together with MglC, also localize asymmetrically to the cell poles and interact in an intricate pattern involving several positive and negative feedback loops to ensure the localization of MglA at the leading pole (213–220) (Fig. 6C). Because the MglB interacts with RomY with low affinity, the formation of the MglB/RomY GAP complex occurs exclusively at the lagging pole, where the concentration of MglB is high (216). Therefore, high GAP activity is confined to the lagging pole, where it outcompetes the RomR/RomX GEF activity (216) (Fig. 6A). Conversely, the RomR/RomX GEF outcompetes MglB GAP activity at the leading pole (216)(Fig. 6A). As a consequence, MglA-GTP and MglA-GDP accumulate at the leading and lagging poles, respectively (Fig. 6A). MglA activates the two motility systems by interacting with downstream effector proteins. In the T4P system, these effectors include SgmX and FrzS that stimulate T4P extension, and in the gliding motility system, they include MreB, AglZ, AglR, and GltJ (83, 84, 189, 221–224) (Fig. 6B).

The Frz chemosensory system acts on the polarity module and causes an inversion of its polarity (Fig. 6A). The two phosphorylated response regulators FrzZ and FrzX implement this output (202, 225). Interestingly, FrzZ-P localizes to the leading cell pole (201) and FrzX-P at the lagging pole (202) (Fig. 6A); however, it remains to be elucidated precisely how they cause the polarity inversion of the proteins of the polarity module. Nevertheless, several theoretical models have been suggested for their mode of action (202, 219, 226).

## SPATIOTEMPORAL REGULATION OF CELL DIVISION IN *M. XANTHUS*

In addition to the spatiotemporal regulation of its movement, *M. xanthus* has emerged as an important model system to understand the spatiotemporal regulation of cell division as well as chromosome organization and segregation. Much of this work was inspired by the surprising discovery that myxospores are diploid, while growing cells contain one to two copies of the chromosome (227). These findings not only suggested that replication is initiated once per cell cycle and completion of replication is followed by cell division in growing cells, but also that cell division is specifically inhibited during myxospore morphogenesis.

A fascinating aspect of cell division in bacteria is that the proteins of the divisome, which is the macromolecular cytokinesis complex that is modulated by the GTP-dependent polymerization of the key cell-division protein FtsZ (228), are largely conserved, but the regulatory systems that correctly position the (Fts)Z-ring are not. These regulatory systems can be divided into those that negatively regulate Z-ring formation throughout the cell except at midcell, for example, the MinC/D/E system in *E. coli* and the MipZ/ParB system in *Caulobacter crescentus* (229), and positively acting systems that directly stimulate Z-ring formation at the nascent division site, for example, MapZ in *Streptococcus pneumoniae* and the *M. xanthus* PomX/Y/Z system (230, 231).

**Fig 7:**
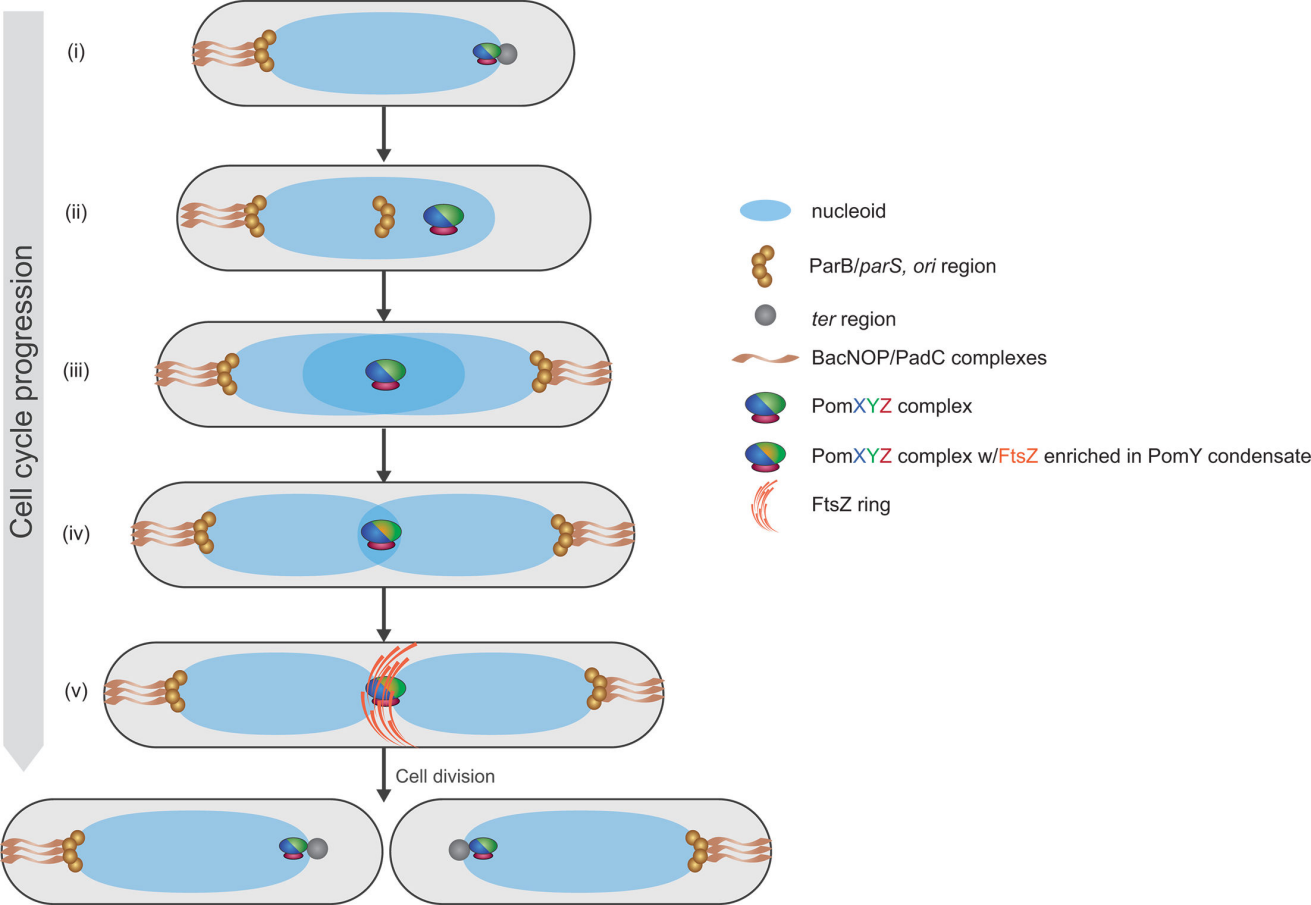
Schematic of the dynamic localization of the PomXYZ complex on the nucleoid and the organization of the *M. xanthus* chromosome segregation machinery over the cell cycle. The schematics follow a cell over the cell cycle starting immediately after cell division (i). (ii–iv) As the cell cycle progresses, the PomXYZ complex translocates across the nucleoid to midcell; in parallel, chromosome replication and segregation proceed, resulting in a chromosome arrangement in which the ParB/*parS* complexes localize in the subpolar regions attached to the bactofilin/PadC complexes, and the *ter* regions around midcell. (v) At midcell, the PomXYZ complex recruits FtsZ and stimulates FtsZ polymerization, resulting in the formation of the Z-ring. FtsZ directly or indirectly recruits all other proteins required for cytokinesis.

The rod-shaped *M. xanthus* divides at midcell. The serendipitous finding that PomZ, a member of the large family of DNA-binding ParA/MinD ATPases, is important for positioning the cell division site at midcell kick-started the detailed analysis of cell division in *M. xanthus* (232). PomZ is encoded in a conserved gene cluster that also encodes PomX and PomY, two proteins that are rich in protein–protein interaction domains and also involved in the regulation of cell division (231). The Pom proteins form a single megadalton-sized, non-stoichiometric nucleoid-associated complex per cell, in which PomY undergoes surface-assisted phase separation on the PomX scaffold to form a biomolecular condensate (231, 233). The DNA-binding PomZ ATPase associates with the PomX/Y complex and the nucleoid (231). Importantly, PomX and PomY both stimulate PomZ’s ATPase activity, resulting in the translocation of the PomX/Y/Z complex across the nucleoid (231, 234). This translocation process ultimately results in the localization of the PomX/Y/Z complex at the middle of the nucleoid mass, which coincides with the midcell. At midcell, the PomY condensate enriches FtsZ to form the Z-ring (231–233) (Fig. 7).

During cytokinesis, the PomX/Y/Z complex undergoes a remarkable fission event, and each daughter cell acquires a smaller complex that associates with the nucleoid close to the new cell pole (Fig. 7). In the ensuing cell cycle, the PomX/Y/Z complex accretes additional PomX/Y/Z molecules while translocating across the nucleoid to midcell (231, 233). This fascinating translocation process has been extensively analyzed to understand how the PomX/Y/Z complex self-organizes to localize precisely at midnucleoid (231, 235). Paradoxically, while we have a good understanding of how cell division is regulated in growing *M. xanthus* cells, we still do not know how cell division is inhibited during myxospore morphogenesis.

To generate appropriately sized daughter cells with the correct chromosome number, accurate positioning of the division site and correct chromosome segregation are essential. In nascent *M. xanthus* cells, the circular chromosome has a unique arrangement and is organized along the longitudinal axis with the *ori* and *ter* regions localized in the subpolar regions of the old and new cell pole, respectively, and the bulk of the chromosome arranged in-between (Fig. 7). Upon initiation of replication immediately after cell division, one *ori* copy segregates to the opposite subpolar region, followed by the rest of the chromosome, while one remains in the original subpolar region (236) (Fig. 7). Chromosome segregation crucially depends on the widely conserved ParA/B/*S* system (236–238). A peculiarity of *M. xanthus* chromosomal organization is that the ParB/*parS* complex is localized in the subpolar region rather than directly at the cell pole as observed in other bacteria (e.g., *C. crescentus*). This peculiar localization is mediated by the filament-forming bactofilins BacN, BacO, and BacP together with the CTP-binding PadC protein, which jointly form a filamentous structure in the subpolar region and interact with ParB at the pole-distal end (239–241) (Fig. 7).

## FRIEND OR FOE: KIN RECOGNITION THROUGH OUTER-MEMBRANE EXCHANGE

**Fig 8:**
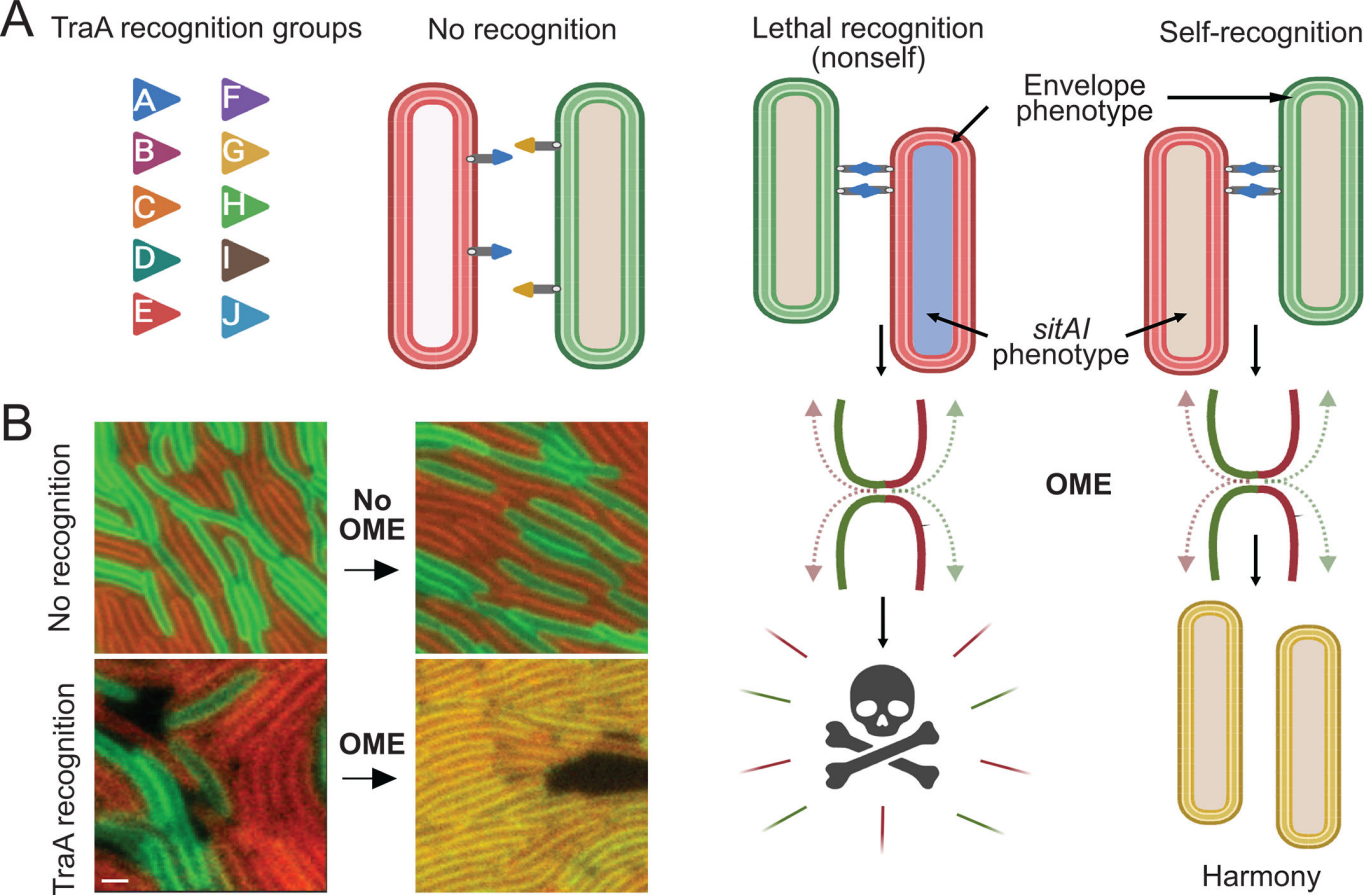
Model of kin recognition and outer membrane exchange (OME). (**A**) 10 experimentally determined TraA recognition groups (colored triangles) govern partner binding. Colored cell envelopes represent OM phenotypes, for example, fluorescent proteins, while colored cytoplasm represents distinct genotypes, particularly in reference to the suite of *sitAI* loci. Following TraA homotypic binding, cells transiently fuse OMs and exchange cargo, including SitA lipoprotein toxins. Clonal cells express cognate immunity and homogenize their OMs (same color), while nonclonal cells are poisoned. (**B**) Merged fluorescent micrographs of *M. xanthus* cells expressing Lipo-GFP or Lipo-mCherry in the periplasmic leaflet of their OMs. 90 min following plating (right), cells that recognize and undergo OME contain both markers, while no OME results in a distinct phenotype. Courtesy of Sheila Walsh; scale bar, 2 µM.

While the differentiation of cell fates and the generation of higher-order structures are both key tenets of true multicellularity, the ability to recognize kin cells is fundamental to these community functions. Kin recognition, as it enables cooperation among genetically related cells while reducing exploitation by non-kin, fostering the stability and functionality of complex multicellular assemblies, is hence a foundation of multicellularity. To transition from solitary, single-cell life into cooperative, multicellular structures, myxobacteria must identify kin to form social groups. The best-studied kin recognition system involves a process called OM exchange (OME) (Fig. 8A), which employs a polymorphic cell-surface receptor known as TraA (242). TraA mediates recognition through homophilic binding, where its variable domain determines the specificity of recognition (243). There are likely well over a hundred different TraA recognition groups in nature. TraA localizes to the cell surface by the type II secretion system and is anchored in the OM by its MYXO-CTERM sorting tag, which is likely lipidated at an invariant cysteine residue (244). TraB (the ORF for which overlaps with *traA*) contains an OM β-barrel and OmpA cell wall-binding domains (245). Together, TraA and TraB form a functional receptor. Following TraA–TraA recognition and cell–cell adhesion, myxobacteria undergo OME, where OM proteins and lipids are transferred between cells. This exchange is rapid, robust, occurs serially between adjacent cells, and involves hundreds of different proteins as well as LPS (246, 247). Since OME is bidirectional and involves lipids, it likely occurs through transient OM fusion, with TraAB functioning as fusogens (245, 248).

The outcomes of OME vary and can result in beneficial cooperation or discriminatory behaviors, at least under laboratory conditions (242). One beneficial behavior involves the rescue of mutant phenotypes, such as motility, via complementation of missing/non-functional proteins (described above). Since DNA is not exchanged, the rescue is transient. In other examples, damaged OMs in one population can be replenished and repaired through OME from a healthy population (249). In addition, cells adapted to environmental stresses, such as detergent resistance, can transfer these beneficial traits to naïve kin via OME (250). In these cases, donor populations also benefit from their seemingly altruistic acts because they help form larger, more fit populations that are better equipped to function in multicellular tasks, such as development, which require a threshold population size (251) (Fig. 8B).

Among the cargo transferred during OME are polymorphic lipoprotein toxins, known as SitA toxins (swarm inhibition toxins), which play a key role in kin discrimination. These toxins belong to six distinct families, with each toxin’s cognate immunity gene located immediately downstream (252). The immunity factors are not exchanged, meaning clonemates are resistant to these toxins, while divergent strains are susceptible. The *sitAI* genes are highly abundant, with myxobacteria genomes containing between 15 and more than 80 loci. These loci are often found within mobile genetic elements, such as plasmids, prophages, and transposons, which promote the expansion and retention of these selfish elements (253). These elements in turn facilitate HGT, leading to rapid social diversification into distinct myxobacterial social groups (254) (Fig. 8A). Therefore, kin recognition through OME occurs in two steps; after TraA–TraA homophilic binding, cells further verify that the partner is clonal through the exchange of polymorphic toxins. Given the number and diversity of exchanged toxins, this secondary step excludes many genotypes with which a focal genotype cooperates in the wild. In doing so, it may help ensure that social groups remain closely related, preventing many non-kin genotypes (including distantly related cheater cells) from causing social disharmony (Fig. 8A). In addition, myxobacteria use other kin-discrimination systems, such as the type VI secretion system, which discriminates against a broader range of distant strains and species of myxobacteria (255). Together, these systems ensure that swarms and fruiting bodies are composed of highly related cells.

## KILLER COHORTS: *M. XANTHUS* ON THE HUNT

Conversely, the opposite of kin recognition is the ability to distinguish and eliminate foreign cells. *M. xanthus* is a renowned predator with specialized mechanisms to feed on other microorganisms: prey cells are killed and lysed, and the released biomass serves as a source for carbon and energy. A multi-factorial predation strategy allows *M. xanthus* to prey on Gram-positive and Gram-negative bacteria, as well as eukaryotes, like yeasts and fungi (256, 257). This fascinating behavior has been the focus of numerous studies, only a fraction of which can be cited here—for comprehensive reviews, please see (258, 259).

Early reports focused on the isolation of bacteriolytic proteins, which *M. xanthus* secretes into the environment to disintegrate nearby prey cells (256, 260). Various lytic activities were detected, but it took several decades to identify some of the responsible proteins. In addition, several bactericidal and antifungal antibiotics were characterized as part of the *M. xanthus* predatory arsenal (256, 261). Some bacteriolytic proteins and antibiotics are released in OM vesicles (262). Notably, the secreted predation factors vary in their efficacy against different prey, and their combined action is needed to target a broad spectrum of microorganisms.

**Fig 9:**
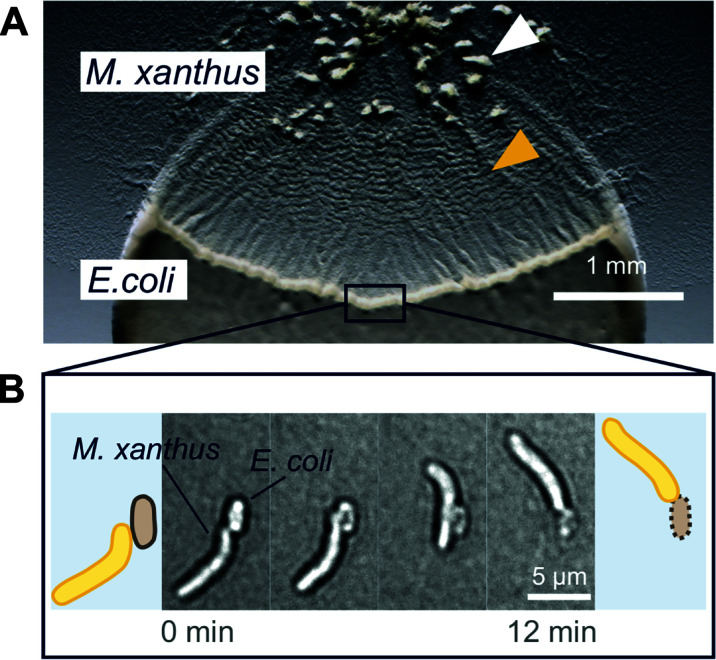
*M. xanthus* is an active predator. (**A**) An *M. xanthus* population (top) invades an *E. coli* colony (bottom), kills, and lyses prey cells and consumes the released biomass. The white arrow denotes fruiting bodies, and the yellow arrow highlights rippling movement by the preying population. (**B**) An individual *M. xanthus* cell approaches *E. coli*, stops upon cell contact, then kills and lyses the prey cell within minutes. Adopted from Reference (265).

The relationship between predation and motility has been extensively studied. *M. xanthus* requires gliding motility and cell reversals to establish contact with prey cells and to assemble multicellular swarms. Swarms approach and invade prey colonies using T4P-dependent motility (Fig. 9A). Hence, the ability to move correlates with predation efficiency (263, 264). Rippling was studied as potentially predation-specific behavior, but it can be observed during development or in the absence of prey as well. Importantly, these studies supplied useful techniques to characterize predation phenotypes, such as the side-by-side spotting of *M. xanthus* and prey populations (208) or a prey lawn assay that links predation efficiency to swarm expansion (257, 263).

The formation of multicellular swarms (dubbed “wolf packs”) was long considered a strict requirement for predation, presumably to ensure that secreted lytic factors accumulate to effective local concentrations (266). Conversely, it was shown that individual *M. xanthus* cells can kill prey via direct cell contact (267, 268) (Fig. 9B). This behavior was recently revisited and attributed to two protein secretion systems, the Tad-like Kil complex and a needle-less Type III secretion system (T3SS*) (269, 270). Both systems transiently accumulate at the predator–prey interface to induce prey killing-and-lysis.

The reciprocal interaction of *M. xanthus* with prey bacteria has repeatedly been a topic of interest. While easy-to-handle *E. coli* was the prey organism of choice in many studies, others focused on soil bacteria. Several species display induced responses to the *M. xanthus* attack, for example, the production of inhibitory antibiotics or protective slime. The upregulation of metal-scavenging siderophores reflects competition for trace elements in predator–prey co-cultures (271). Moreover, other abiotic factors, like temperature, may even invert the predator–prey relationship in some cases (272). On a broader scale, it was demonstrated that predatory Myxococcota are the most abundant micro-predators in soil environments, highlighting the importance of myxobacterial predation for the microbial food web across diverse ecosystems (273).

## SHEDDING LIGHT ON CAROTENOID DEFENSE IN THE MYXOBACTERIA

Even in the absence of antagonistic cells or compounds, myxobacteria can protect themselves from things as seemingly innocuous as light. In the 1960s, it was discovered that *M. xanthus* colonies grown continuously under light turn reddish owing to carotenoid synthesis, indicating a photoprotective carotenogenic response that may confer a fitness advantage in its natural soil habitat (274, 275). It was not until the development of a variety of genetic tools in the 1980s that in-depth studies of the *M. xanthus* carotenogenic response were rekindled and continue to this day, making use of color changes as powerful visual tools for genetic analysis. The striking distribution of particular *M. xanthus* factors involved in photoreception and signal transduction among myxobacteria and eukaryotes, but not in other bacterial phyla, adds to growing evidence supporting a theory on the origin and early evolution of eukaryotic cells that posits involvement of an early myxobacterium (276, 277).

Blue light triggers a genetic program that activates the expression of a regulatory operon encoding proteins CarQ, CarR, and CarS, a large genomic cluster comprising nine structural genes and two paralogous regulatory genes (*carA* and *carH*), and an unlinked structural gene (*carC*/*crtIb*) (278–280). CarQ stands out as one of the earliest identified extracytoplasmic-function (ECF) σ factors. Light liberates CarQ from its membrane-associated anti-σ factor CarR, and free CarQ associates with RNA polymerase to directly activate *crtIb* and its *carQRS* operon (281, 282). The membrane protein CarF mediates the signaling by singlet oxygen (^1^O_2_), an extremely harmful species produced upon blue-light exposure that somehow provokes CarR inactivation (283).

The homologs of CarF, largely restricted to myxobacteria and animals, including humans, turned out to be the long-sought lipid desaturase involved in the biosynthesis of plasmalogens, a special type of glycerophospholipid with unique physicochemical properties that have been linked to various human pathologies, including cancer and neurodegenerative diseases (284). The story of CarF thus illustrates how the pursuit of basic bacterial research helped to unearth a human protein with a crucial, previously unknown function, thereby opening a door to therapeutic applications.

Transcriptional activation by CarQ also requires a unique global regulatory complex of CarD and CarG. CarD stands out as the only prokaryotic protein with a hallmark DNA-binding domain resembling eukaryotic HMGA (High Mobility Group A) proteins, which are crucial factors in enhanceosome function (285). Besides CarQ, many of the approximately 45 ECF σ factors in *M. xanthus*, including DdvS, require the CarD/G complex to function. DdvS is the first ECF σ that controls the expression of an antiphage defense island comprising two cyclic oligonucleotide-based antiphage signaling systems and a type III-B CRISPR-Cas system (286, 287).

The discovery of a second pathway came from the observation that the C-terminal oligomerization domain of CarH shared sequence similarity to the B_12_-binding domain typically found in enzymes. CarH relies on coenzyme B_12_ or adenosylcobalamin to sense light (UV, blue, green) and to modulate its oligomerization state, DNA binding, and repressor activity, thereby providing a direct pathway to regulate light-induced carotenogenesis (288). This pathway has since been shown to be widespread across bacteria and appears to be the ancestral one in myxobacteria (289, 290). Discovery of CarH and its mode of action heralded the emergence of a new photoreceptor family and a new facet of vitamin B_12_ and continues to inspire various ingenious applications in optogenetic and light-controlled synthetic biology (291–293).

## LIGHT AND MULTICELLULAR DEVELOPMENT IN MYXOBACTERIA

Besides triggering the photoprotective carotenogenic response, light is known to affect the development of certain myxobacteria. Exposure to light is required for fruiting-body development of *S. aurantiaca* (294–296), which contains two photoreceptors, bacteriophytochromes (BphPs), that are absent in *M. xanthus* (296–298). While BphPs in photosynthetic bacteria modulate the synthesis of light-harvesting complexes (299–301), their physiological function in non-photosynthetic bacteria remains largely unknown (302). Both BphPs from *S. aurantiaca* contain histidine kinase domains (HK) that are regulated by red and far-red light (303). When incubated on starvation media, *S. aurantiaca* only forms fruiting bodies under far-red light (296).

The photomorphogenic response in *S. aurantiaca* led to the hypothesis that a BphP also regulates the development of *Myxococcus macrosporus* (the closest genetic relative to *M. xanthus*) that contains one BphP-encoding gene with a C-terminal HK domain (298, 304). Light regulates the expression of BphP in *M. macrosporus* (305). Interestingly, the fruiting bodies of *M. macrosporus* formed concentric rings in the dark over 14 days, resembling oscillatory growth patterns of the fungus *Neurospora crassa*. Exposure to red and far-red light disrupted this rhythmic or circadian-like phenotype, suggesting a possible role for BphP (305, 306). The first bacterial circadian clock, demonstrated in cyanobacteria, comprises KaiA, KaiB, and KaiC proteins (307). Homologs of KaiB and KaiC proteins are found in many bacteria, including Myxococcales (305). As some myxobacteria encode BphPs in tandem with KaiC genes, circadian rhythm could regulate fruiting-body formation. In fact, *M. macrosporus* HW-1 isolated from coastal microbial mats demonstrates circadian rhythmicity in its gene expression (308).

## SECONDARY METABOLITES OF *M. XANTHUS* AND OTHER MYXOBACTERIA

Up to 11% of the extraordinarily large *M. xanthus* genome is related to the biosynthesis of secondary metabolites. So far, we can only deduce such metabolites through chemical characterization in a limited number of cases. Secondary metabolites identified from *M. xanthus* and other myxobacteria often display large structural varieties and intriguing biological activities (309). These complex molecules are usually produced by multi-enzyme systems that consist of polyketide synthases, non-ribosomal peptide synthetases, and their combinations. Low-molecular-weight building blocks are linked in a highly variable manner and further modified by additional specific enzymes either during or after the synthesis of the molecular scaffolds (310). Besides polyketide synthase- or non-ribosomal peptide synthetase-borne secondary metabolites, other types have also been reported, including homospermidines or the ribosomally synthesized and post-translationally-modified cittilins and crocagins (311–313).

The first myxobacterial secondary metabolite, the antifungal ambruticin produced by certain strains of *S. cellulosum*, was discovered in 1977 (314, 315). To date, more than 130 classes of secondary metabolites have been described from myxobacteria. Many of them comprise families of up to 20 derivatives from common scaffolds. These compounds show antibacterial/antifungal/cytotoxic activities, act as iron chelators, or contribute to at least one of the extraordinary capabilities of myxobacteria, including development, predation, and swarm spreading (309, 310, 316).

The high energetic costs for the biosynthesis of these natural compounds reflect the benefits they confer in the natural soil habitats of myxobacteria, which usually lack free nutrients. The production of ambruticin is a good example: due to its inhibitory effect on *M. xanthus* fruiting-body formation under nutrient-poor conditions, ambruticin-producing *S. cellulosum* may outcompete *M. xanthus* in the soil environment (317). Other identified myxobacterial secondary metabolites are likely to offer similar advantages as their production is transcriptionally linked to swarming, development, or predation (318). Among these are the antifungal myxalamids (319), the antibiotic myxovirescin that kills prey (261, 320), the iron chelator myxochelin (321), DKxanthenes that are required for efficient fruiting-body formation and sporulation (322), and homospermidines that are exclusively produced during development (311). By contrast, the functions of various secondary metabolites such as myxoprincomides remain to be determined (323). In addition, numerous biosynthetic gene clusters within myxobacterial genomes appear to be “silent,” as their corresponding products remain unidentified.

Despite 50 years of intense research, we have only discovered the tip of the iceberg. In the era of genomics, as the number of novel species, genera, and families rises steadily, it is apparent that the full biosynthetic potential of myxobacteria has yet to be revealed (324, 325). Laboratory conditions that lack the respective stimuli for production might prevent the identification of many secondary metabolites that have therapeutic activities. Nevertheless, the discovery of novel myxobacterial anti-infectives such as cystobactamids, disciformicins, thiamyxins, pyxidicyclines, and alkylpyrones has demonstrated the value of genome- and biodiversity-driven approaches (326–330).

## ADAPTING AND THRIVING: MYXOBACTERIAL EVOLUTION AND ECOLOGY

As detailed above, myxobacteria are versatile and effective model systems for interrogating a wide range of processes, starting from the level of specific molecules up to macroscopic groupings of cells. On an even grander scale, an important aspect of myxobacterial research is the endeavor to understand how ecological forces—whether abiotic, social, or community—influence the origin, evolutionary construction, diversification, and character of myxobacterial social and predatory behaviors, and reciprocally how myxobacteria shape the ecology and evolution of other organisms. Through analysis of natural population diversity, experimental ecology, and experimental evolution, progress has begun to be made toward this goal.

Myxobacterial fruiting-body diversity is stunning (Fig. 1) and begs for an evolutionary explanation. Exploration of myxobacterial diversity was greatly advanced by, among others, the wife-and-husband team of Helena Krzemieniewska and Seweryn Krzemieniewski, who extensively sampled Polish myxobacteria in the 1920s and 1930s (331), and later in the last century by Wolfgang Dawid (332), who broadly isolated myxobacteria across several continents, including Antarctica. Such earlier work ultimately led to current efforts by multiple groups to understand global patterns of genomic and phenotypic diversity (both terrestrial and aquatic) and how they are shaped by ecological context. Comparative analysis of interspecific genomic diversity has begun to reconstruct the evolutionary steps by which myxobacterial traits, including motility (180) and fruiting-body development (19, 98), previously evolved and diversified. To allow finer-scale analysis of natural diversity and evolution—for example, how *M. xanthus* predation profiles (257) or kin-group identities diversify (333)—biogeographically explicit and fine-scale analysis of *M. xanthus* intraspecific diversity was initiated two decades ago (334) and continues expanding today.

Well before quorum sensing was discovered in other bacteria, crucial roles of high cell density in enhancing growth from extracellular metabolism of complex molecules (335) and triggering complex social behaviors were demonstrated in experiments with *M. xanthus* (15). Such early experiments manipulating ecological variables established a growing tradition of experimental ecology that seeks to understand how ecological context and myxobacterial traits interact, for example in differential advantages of distinct motility systems across surface types (336), what conditions can induce and allow fruiting-body development (295), or how temperature can determine whether *M. xanthus* acts as predator or prey (272). Martin Dworkin played a major early role in identifying important ecological questions (15), many of which remain inadequately answered and thus continue to fuel the field today. These include questions about the roles of myxobacteria as keystone taxa in nutrient cycling through scavenging and predation (273), how variable environmental conditions interact with different aspects of myxobacterial life cycles, and the character of interactions between distinct members of the same species (which can be both antagonistic and synergistic (337)) and between different species.

In 1984, Zahavi and Ralt wrote, “It will be hard to consider any better candidate among bacteria than myxobacteria for testing theories concerning the evolution of social adaptation” (15). Their statement foreshadowed the rise of *M. xanthus* over the past three decades as a model system for direct experimental study of how microbial social behaviors and predator–prey interactions evolve in real time. As summarized at myxoee.org (338), evolution experiments with myxobacteria (MyxoEEs) have led to the discovery of (i) “cheating” and “policing”-like behaviors in bacteria, (ii) frequency-dependent fitness-rank reversal as a mechanism of cooperation maintenance, (iii) *de novo* origins of kin discrimination, (iv) novel forms of social motility, (v) how different types of social interaction can drive multicellular diversification, (vi) mechanisms of predator–prey co-adaptation, and have (vii) additionally tested hypotheses emerging from optimal foraging theory. They have also uncovered previously unknown early genetic regulators of fruiting-body development (339), illustrating how analysis of experimentally evolved lineages can generate important contributions to mechanistic molecular biology. Going forward, ongoing integration of the research approaches highlighted here with theoretical modeling, increasing understanding of myxobacterial molecular biology, and better approaches for studying microbes in their natural habitats will make the coming years an exciting period for eco-evolutionary studies of myxobacteria.

## PERSPECTIVES

The study of myxobacteria has advanced significantly over the past few decades. While previous questions continue to be resolved, fresh research topics are also emerging, which will undoubtedly shape the future of the myxobacterial field:

### Synthetic biology and genetic engineering

The growing understanding of myxobacterial genetic systems, particularly their complex secondary metabolite production, provides fertile ground for advancing synthetic biology and genetic engineering. The large and diverse genomes of myxobacteria offer untapped reservoirs for novel natural products. Future research may focus on optimizing the biosynthesis of secondary metabolites, including previously “silent” gene clusters, for use in drug development, agriculture, and biotechnology. Moreover, the ability to engineer myxobacterial motility and social behavior could enable the design of synthetic microbial communities with tailored functionalities.

### Evolution and ecology of social behaviors

The evolution of social behaviors remains a cornerstone of myxobacterial research. Future studies could expand on the regulation of multicellular development and the evolution of group-level behaviors. Investigating the co-evolution of myxobacteria with their microbial prey, the dynamics of predator–prey interactions, and the genetic and environmental factors that influence kin recognition will provide broad insights into the evolution of social behaviors in microorganisms. Myxobacteria are key players in soil ecosystems, often interacting with other bacteria and eukaryotes. As the field of microbiome research expands, understanding the role of myxobacteria in shaping microbial communities will become increasingly important. Future work may focus on how myxobacteria interact with other soil microorganisms, contribute to nutrient cycling, and influence the microbiomes of plants and animals. Given their remarkable abilities in eliminating other microorganisms and degrading complex organic molecules, myxobacteria hold great promise in environmental remediation and biocontrol.

### Physiology and stress responses

The ability of myxobacteria to adapt to various environmental stresses is another exciting area of research. Their tolerance and resistance to antibiotics, ability to survive in nutrient-poor environments, and photoprotective responses offer valuable insights into microbial survival strategies. Future research could investigate the molecular mechanisms behind these stress responses and their potential applications in biotechnology, such as the development of stress-tolerant microbes for industrial processes.

### Harnessing advanced analytical tools

The continued development of high-throughput sequencing, mass spectrometry, and imaging technologies will be instrumental in uncovering the full complexity of myxobacterial biology. For example, single-cell transcriptomics and proteomics could provide unprecedented insights into the heterogeneity of myxobacterial populations, revealing how different cells within a community contribute to group-level behaviors. In addition, advancements in microscopy techniques, such as super-resolution imaging and live-cell tracking, will allow researchers to visualize and quantify the dynamics of myxobacterial motility, development, and social interactions in real time.

## CONCLUSION

Microorganisms exhibit remarkable species richness and diversity in their size, shape, habitat, metabolism, subcellular organization, and the structures they form. Moreover, they have essential roles in various ecosystems as well as in human, animal, and plant health. Microbiome studies have taught us that the standard model organisms for studying microbial cell function and metabolism are rarely—if ever—the most abundant in a particular ecosystem or habitat. To truly grasp microbial structure, function, ecology, and evolution, we must look beyond just a handful of popular model organisms. A broader perspective is essential for deeper insights. Through *M. xanthus*, we have not only learned that different species have evolved different solutions to the same problem (e.g. regulation of motility and cell division), but also learned entirely new biology (e.g. mechanisms underlying kin recognition, outer membrane exchange, predation, and secondary metabolite biosynthesis). With its remarkable complexity and ease of handling, *M. xanthus* will continue to serve as a versatile and elegant organism for exploring fundamental bacterial functions, multicellular behaviors, and microbial evolution. As we look ahead to the years to come, we anticipate groundbreaking discoveries that will not only enhance our understanding of myxobacteria but also propel advancements across the entire field of microbiology.
